# Supplementary material for: Role of lean leadership in the lean maturity—second-order problem-solving relationship: a mixed methods study
Source: BMJ Open. 2019 Jun 3;9(6):e026737. doi: 10.1136/bmjopen-2018-026737 (PMC6561417; doi:10.1136/bmjopen-2018-026737)
Supplement: Author's manuscript [file bmjopen-2018-026737.draft_revisions.pdf]

BMJ Open is committed to open peer review. As part of this commitment we make the peer review history of every article we publish publicly available.

When an article is published we post the peer reviewers' comments and the authors' responses online. We also post the versions of the paper that were used during peer review. These are the versions that the peer review comments apply to.

The versions of the paper that follow are the versions that were submitted during the peer review process. They are not the versions of record or the final published versions. They should not be cited or distributed as the published version of this manuscript.

BMJ Open is an open access journal and the full, final, typeset and author-corrected version of record of the manuscript is available on our site with no access controls, subscription charges or pay-per-view fees (http://bmjopen.bmj.com).

If you have any questions on BMJ Open's open peer review process please email info.bmjopen@bmj.com

BMJ Open

The role of lean leadership in the lean maturity – second-order problem-solving relationship: a multiple case study

Journal:	*BMJ Open*
Manuscript ID	bmjopen-2018-026737
Article Type:	Research
Date Submitted by the Author:	17-Sep-2018
Complete List of Authors:	Bijl, Arie; University of Groningen, Faculty of Economics and Business, Department of Operations,<br>Ahaus, Kees; University of Groningen, Faculty of Economics and Business, Department of Operations,<br>Ruël, Gwenny; University of Groningen, Faculty of Economics and Business, Department of Operations,<br>Gemmel, Paul; Ghent University, Faculty of Economics and Business Administration, Department of Innovation, Entrepreneurship and Service Management,<br>Meijboom, Bert; Tilburg University, Department of Tranzo
Keywords:	Lean leadership, Lean maturity, second-order problem solving

SCHOLARONE™
Manuscripts

For peer review only

The role of lean leadership in the lean maturity – second-order problem-solving relationship: a multiple case study

Arie Bijl
Department of Operations
Faculty of Economics and Business
University of Groningen, The Netherlands
Nettelbosje 2
9747 AE Groningen, The Netherlands
T. +31503637078
Email address: ariebijl94@gmail.com

Kees Ahaus **(corresponding author)**
Department of Operations
Faculty of Economics and Business
University of Groningen, The Netherlands
Nettelbosje 2
9747 AE Groningen, The Netherlands
T. +31503637078, M. +31651591859
Email address: c.t.b.ahaus@rug.nl

Gwenny Ruël
Department of Operations
Faculty of Economics and Business
University of Groningen, The Netherlands
Nettelbosje 2
9747 AE Groningen, The Netherlands
T. +31503637078
Email address: g.c.ruel@rug.nl

Paul Gemmel
Department of Innovation, Entrepreneurship and Service Management
Faculty of Economics and Business Administration
Ghent University, Belgium
Email address: Paul.Gemmel@ugent.be
Tweekerkenstraat 2
9000 Gent, Belgium
T. +3292643518

Bert Meijboom
Department of Tranzo
Tilburg University, the Netherlands
Email address: B.R.Meijboom@uvt.nl
Postbus 90153
5000 LE Tilburg, The Netherlands
T. +31134663237

For peer review only

The role of lean leadership in the lean maturity – second-order problem-solving relationship: a multiple case study

ABSTRACT

Objectives: To investigate the relationship between Lean adoption and problem-solving behaviour in nursing teams, and to explore the practices of Lean leaders in nursing wards to reveal how they can stimulate second-order problem-solving within their teams.

Design: A mixed-methods retrospective multiple case study using semi-structured interviews supplemented by document research. Interview data were used to assess the level of Lean maturity (based on a customized validated instrument) and the level of second-order problem solving (based on scenarios). Within-case and cross-case analyses were employed to identify Lean leadership practices.

Setting: 14 nursing teams, with different levels of Lean maturity, in one Dutch hospital.

Participants: Three members of each nursing team were interviewed: the team leader, one nurse from the ward's core team for the Lean-based quality improvement programme and one nurse outside the core team. Further, the programme coordinator was interviewed resulting in a total of 43 interviews.

Interventions: The nursing teams were in various phases of a Lean-based quality improvement programme: 'The Productive Ward – Releasing Time to Care'.

Results: A highly significant positive relationship between Lean maturity and second-order problem solving was found: $\beta = 0.68$, $R^2 = 0.46$, $p < 0.001$. Further, the results indicated a potential strengthening effect of Lean leadership on this relationship. Seven Lean leadership practices emerged from the data collected in nursing ward settings: (1) convincing and setting an example, (2) unlocking individual and team potential, (3) solving problems systematically, (4) enthusing, actively participating and visualizing, (5) developing self-managing teams, (6) sensing, as orchestrator, what is needed for change and (7) listening, sharing information and appreciating. These practices have a strong link with transformational leadership.

Conclusions: As Lean matures, nursing teams reach a higher level of second-order problem solving. In later stages, Lean leaders increasingly relinquish responsibility by developing self-managing teams.

Keywords: Lean leadership, Lean maturity, second-order problem solving

Word Count: 4,172

Strengths and limitations of this study

- The study fills a gap in knowledge as to whether more-mature Lean teams show a higher level of second-order problem-solving behaviour.
- In addition, it provides valuable insight into typical Lean leadership practices and how these are linked to mainstream leadership theories such as transformational leadership theory.
- Future research attempting to measure Lean maturity in healthcare environments may benefit from using the new, validated scale developed, which incorporates six

For peer review only - http://bmjopen.bmj.com/site/about/guidelines.xhtml

items from Malmbrandt and Ahlström's instrument[1] that were found to be relevant in nursing wards.

- Another strength of the study is its use of Gioia methodology[2] to analyse inductively the Lean leadership data by giving 'informant-centric', *in vivo* first-order codes, by organizing these into second-order concepts that are finally linked to seven aggregated Lean leadership dimensions.
- The nursing teams all came from one Dutch hospital. However, this choice had the benefit that it provided nursing teams with a range of Lean maturities working under similar circumstances.

Introduction

The earliest applications of Lean thinking in the healthcare sector were more than a decade ago. Since then, Lean has gained in popularity in the sector.[3] The primary goals of Lean in healthcare have been to increase the quality of care and to increase efficiency.[4] To achieve these goals, most healthcare organizations have emphasized the application of Lean tools to reduce direct waste, but neglected developing the problem-solving abilities of frontline employees.[5] This approach may have created some process improvements, but long-term hospital-wide benefits have rarely been achieved.[6]

To realize Lean's greater potential, it is often suggested that structured problem solving should be developed throughout the organization to improve processes in a sustainable way.[5, 7] One well-known and effective approach to this is *second-order problem solving.* This involves the in-depth questioning of work practices to uncover and remove the root causes of problems.[8] This approach is in sharp contrast with *first-order problem solving* where problems are resolved in an ad-hoc manner, while underlying causes remain.[9] A recent study by Gemmel *et al.*[5] suggests that second-order problem solving was more prevalent than first-order problem solving in nursing teams with high levels of Lean maturity. However, evidence from a larger sample of wards is needed to confirm this.

Earlier studies have suggested that team leaders could stimulate second-order problem-solving behaviour by nurses.[8] Team leaders are crucial in creating and sustaining the benefits of Lean adoption in hospital wards, since they can help to create a culture of continuous improvement, empower frontline employees and foster participation.[10, 11] Although the literature often emphasizes the importance of Lean leadership, there are few empirical studies of Lean leadership in healthcare.[11, 12] Moreover, previous studies have not connected Lean leadership with other leadership theories.[11] Consequently, an in-depth empirical study of Lean leadership in nursing wards is needed, whereby the identified leadership practices can be positioned within existing leadership approaches in order to enhance our understanding of this concept.

In this paper, we study the relationship between Lean maturity and second-order problem-solving behaviour. In addition, we explore the meaning attached to Lean leadership in nursing wards and discover how Lean leadership moderates the Lean maturity – second-order problem-solving relationship. In our study of the Lean leadership concept, we focus solely on identifying leadership practices in nursing wards (i.e. in terms of team leadership). We answer the following research questions:

- How does Lean maturity affect the problem-solving behaviour of nursing teams?
- What constitutes Lean leadership in nursing wards?
- How does Lean leadership affect the relationship between Lean maturity and second-order problem solving?

For peer review only - http://bmjopen.bmj.com/site/about/guidelines.xhtml

Background

Lean maturity

The concept of Lean maturity needs careful consideration if one is to accurately assess the impact of Lean implementation on the problem-solving behaviour of nursing teams.[5] Malmbrandt and Åhlström[1] argue that the extent of an organization's Lean adoption can be measured using an instrument that incorporates measures to assess Lean enablers, Lean practices and performance. Lean enablers represent the supporting structure or preconditions of Lean, including the training of employees and dedicating time and resources to improvement work. Lean practices correspond to Lean principles, such as continuous improvement and eliminating waste. Performance refers to the results of Lean adoption in terms of measures such as quality, customer satisfaction and costs. Together, these three dimensions determine the Lean maturity level of an organization. These can range from no adoption through the increase of continuous improvement activities to an exceptional, well-defined and innovative approach.[1]

Second-order problem-solving behaviour

Nurses, as frontline service providers, play an essential role and are in the best position to uncover and eliminate the root causes of problems and, thereby, help their team learn.[8, 9] Second-order problem solving occurs when "the worker, in addition to patching the problem so that the immediate task at hand can be completed (i.e. first-order problem solving), also takes action to address underlying causes"[9(p.63)].

Tucker and Edmondson[8(p.61)] distinguish five broad actions linked to second-order problem solving: (1) communicating to the person or department responsible for the problem, (2) bringing the problem to the manager's attention, (3) sharing ideas about the cause of the situation and how to prevent recurrence, (4) implementing changes and (5) verifying that changes have the desired effect.

Nurses often apply first- and second-order problem-solving approaches. Quick workarounds may be needed if patients cannot wait for the promised care.[13] However, only by applying second-order problem solving can real process improvements be made. Gemmel *et al.*[5] go as far as to state that a learning organization based on second-order problem solving should be one of the ultimate goals of Lean.

Lean leadership

The importance of effective 'Lean leadership' during Lean implementations is widely recognized.[10] A few studies in healthcare have suggested that Lean leadership can be linked to transformational leadership theory.[11, 14] With transformational leadership, followers are motivated to do more than originally expected and to feel trust, loyalty, respect and admiration towards their leader.[15] Transformational leaders develop their followers through four key dimensions. *Idealized influence* refers to a leader being a role model, one who is admired and respected by their followers. *Inspirational motivation* implies that a leader motivates those around him/her and arouses their spirit. Transformational leaders use *intellectual stimulation* to stimulate their followers to be innovative and creative. Finally, a transformational leader attends to the need for achievement and growth of individuals through *individual consideration* by acting as a coach.[15] Transformational leadership is different from transactional leadership. The latter is merely an exchange process to motivate follower compliance, where a leader clarifies performance criteria, states expectations and determines what followers receive in return.[15] The link between transformational leadership theory and Lean leadership

For peer review only - http://bmjopen.bmj.com/site/about/guidelines.xhtml

is relatively under-researched.[11] In healthcare, there are only a few empirical studies investigating Lean leadership practices.[11, 12]

Conceptual model

Figure 1 displays the conceptual model applied in this study. First, in line with Gemmel *et al.*[5], we expect greater Lean maturity to potentially lead to an elevated degree of second-order problem solving in nursing teams. Second, since Lean leaders are seen as essential in enhancing the problem-solving abilities of healthcare staff[10], we expect effective Lean leadership to strengthen the relationship between Lean maturity and second-order problem solving.

Please insert figure 1 here

Methods

This research involves 14 case studies of nursing teams in different departments of a Dutch hospital; each in various phases of a Lean-based quality improvement programme: 'The Productive Ward – Releasing Time to Care' (from now on referred to as PW). This programme trains nursing teams how to apply Lean tools and principles in their daily work and claims to increase the time nurses have for direct patient care.[16] A key tool incorporated in PW is the Hairdryer model. This teaches nurses how to engage in effective problem solving by discussing problems in groups, mapping the current situation, collecting data, implementing changes and assessing their impacts.

Each of the 14 nursing teams has a PW core team that leads the change. Generally, this consists of the team leader and a few nurses. To obtain reliable data, the team leader, one nurse inside and one nurse outside each PW core team were interviewed. Nursing teams with differing periods in the programme (0-24 months) were selected as cases. We expected this to provide us with variation on the Lean maturity variable. Cases were selected with different durations in the belief this would produce different results but for predictable reasons (i.e. theoretical replication).[17] Table 1 shows the selected cases within each duration level.

Table 1 Selected cases within each duration level

Duration	Selected cases
Level 1 (0-6 months)	L, N
Level 2 (6-12 months)	H, I, J, K
Level 3 (12-18 months)	D, E, F, G, M
Level 4 (18-24 months)	B, C
Level 5 (24 + months)	A

Data collection

In total, 43 semi-structured interviews were conducted in December 2016 with an average duration of 40 minutes, a minimum of 24 minutes and a maximum of 68 minutes. More than one researcher was present at each interview. The interview questions related to Lean maturity were designed to measure several Lean enablers and Lean practices. These provided accurate insight into the progress being made with Lean implementation. These Lean enablers and practices were extracted from Malmbrandt and Åhlström's instrument.[1] Their instrument contains 34 items to assess Lean service adoption but we decided to include only six items for several reasons. First, accurate and reliable performance data were lacking for most departments making it impractical to include the performance-oriented items. Further, some items had strong links with

For peer review only - http://bmjopen.bmj.com/site/about/guidelines.xhtml

other main variables, such as management commitment and understanding (Lean leadership) and the degree of structured problem solving (second-order problem solving). Finally, some items were not considered relevant, or else too complex, to measure (such as the levelling and balancing of workloads). The customized instrument included three Lean enablers (employees' understanding of Lean; time and resources allocated to improvement work; bi-directional vertical information flows), and three Lean practices (identification of patient value, workplace design for flow; and visualization of information and improvements).

Second-order problem solving was measured through a set of questions in the form of scenarios derived by Gemmel *et al.*[5] These scenarios describe several types of problems nurses face in their daily work. They were used to obtain information about the actions nurses took when faced with a problem. Nurses were also asked to provide examples of a problem where they engaged in second-order problem solving so we could obtain further insights into this concept.

Lean leadership was studied from various viewpoints with different open and semi-structured interview questions posed to team leaders and nurses. We specifically asked about leadership practices adopted to support and implement the PW programme and to develop the team by applying PW. Questions related to generic leadership practices were avoided.

Data analysis

The interviews were coded by multiple researchers to enhance the reliability of the findings.[17] From each interview, based on the instruments applied, scores were deduced for the levels of Lean maturity and second-order problem-solving behaviour. The scores for each set of three ward members were checked for consistency. This process eventually resulted in a low, medium or high rating, for each team, of its Lean maturity and second-order problem-solving levels.

A correlation analysis was performed to explore whether the duration of the PW programme corresponded with the level of Lean maturity. This revealed a strong correlation ($r = 0.58$; $p < 0.001$). Next, scores were attributed to the three enablers and three practices of Lean on a five-point scale based on the original instrument of Malmbrandt and Åhlström.[1] Although the Lean enablers and practices were extracted from a validated instrument, a confirmatory factor analysis (CFA) was performed to test the unidimensionality of the six selected items. The CFA extracted a single component with all factor loadings above 0.7, indicating the construct validity of measuring Lean maturity using our six-item scale. In addition, the reliability analysis returned a Cronbach's alpha of 0.84 suggesting a strong internal consistency among the Lean enablers and practices.

The information collected through the scenarios was used to link each respondent to a category of first- or second-order problem-solving behaviour. For this, we used a scale with eight levels. Levels 1, 2 and 3 represent first-order problem-solving approaches, whereas levels 4 through 8 represent second-order problem-solving actions as described by Tucker and Edmondson[8(p.61)], with level 8 indicating the highest degree of second-order problem solving.

For the within-case analysis of Lean leadership, all the interviews were analysed to extract useful data. Using the rigorous step-by-step approach proposed by Gioia *et al.*[2], the data were coded inductively by two researchers. This led to a coding tree of Lean leadership practices (available on request) involving 339 *in vivo* codes, 29 themes and 7 aggregate dimensions. The list of themes was then used by two researchers to independently code nine out of 43 interviews (20% of the interviews). A comparison

For peer review only - http://bmjopen.bmj.com/site/about/guidelines.xhtml

demonstrated an acceptably high inter-rater reliability in terms of assessing Lean leadership with a Krippendorff's alpha value of 0.85.[18]

In the cross-case analysis, cases where consistent low, medium or high levels of both Lean maturity and second-order problem solving were found were compared with cases that deviated from this expectation of consistency to determine whether Lean leadership strengthened the relationship between Lean maturity and second-order problem solving.

Patient and public involvement

There was no patient or public involvement in this study. The results of this work were disseminated to representatives of the hospital. Once the study has been published, the main findings will be presented in education of Master students.

Results

The results showed that increased Lean maturity positively influences the level of second-order problem solving in nursing teams. This relationship was tested through a single linear regression analysis. A strongly significant positive relationship was found between Lean maturity and second-order problem solving: $\beta = 0.68$, $R^2 = 0.46$, $p < 0.001$.

Within-case analysis

The in-depth analysis of the meaning of Lean leadership resulted in the identification of seven Lean leadership practices: (1) convincing and setting an example, (2) unlocking individual and team potential, (3) solving problems systematically, (4) enthusing, actively participating and visualizing, (5) developing self-managing teams, (6) sensing, as orchestrator, what is needed for change and (7) listening, sharing information and appreciating. Each of these practices is explained below.

First, Lean leaders helped nurses to become acquainted with the programme. They constitute the driving force of PW by setting an example and actively promoting PW in their team. Initially, many nurses needed to be convinced of the value of PW and, so, an important activity was to explain PW modules and point out potential benefits. These included the time that could be saved by applying PW: *"I try to give insights into why we have to change certain things"* (D-01: in this notation, D refers to team D, participant '01' refers to the team leader; '02' and '03' refer to the nurses inside and outside the PW core team respectively).

Lean leaders also aimed to unlock the greater potential of nurses and their team by encouraging everyone to become active within PW, for instance, by stimulating membership of the PW core team and participation in improvement board sessions. Nurses were also invited to indicate a preferred improvement project, based on their own interests and capabilities, to work on within PW. Within these PW projects, Lean leaders adjusted their own degree of involvement for each individual based on the level of experience of each nurse. This gave room for individual potential to develop.

Further, Lean leaders were involved in systematic problem solving. They were present during team discussions where problems were analysed by applying PW tools. Leaders also encouraged their team to solve problems through the Hairdryer model, as they felt that this model should be incorporated in nurses' work practices. In addition, the leaders cooperated with nurses who wanted to engage in systematic problem solving, as one of the participants stated: *"Our team leader would help us by providing input on how we can approach things"* (C-03).

Another key Lean leadership practice was to enthuse the team, actively participate in PW and visualize. First, the leaders encouraged nurses to organize improvement board

For peer review only - http://bmjopen.bmj.com/site/about/guidelines.xhtml

sessions and to carry out daily evaluations of processes. We observed that core-team members were regularly rotated, as being a member of the PW core team was perceived as a strong motivating factor by nurses. This leadership involvement was seen as highly important by both nurses and leaders, as the following quotes illustrate: *"The leader motivates me through her enthusiasm, and by showing that she wants it herself"* (N-02) and: *"I need to bring along the group through enthusiasm"* (E-01). Finally, many participants pointed out that leaders used visualization through photographs or videos to create awareness of a non-optimal situation and to demonstrate PW accomplishments.

Lean leaders also developed self-managing teams. They encouraged nurses to actively take the lead, for instance, by giving nurses ownership of improving certain processes through PW tools. It was frequently noted that, as the implementation of PW progressed, Lean leaders would increasingly relinquish tasks and responsibility to increase the sense of ownership among the nurses in the team. This interpretation is supported by the following quotes: *"Through this programme, my tasks are diminished as nurses themselves take more responsibility"* (A-01) and *"The leader does not want to keep tight control of everything, he relinquishes tasks and gives guidance, which motivates us to be actively involved in the programme"* (H-03).

Lean leaders continuously thought about what was needed for change to take place. For instance, they facilitated this by providing time and resources to engage in PW activities and they ensured that PW meetings regularly took place. It was also stated that the leaders provided direction and kept an overview of PW's progress within their team, without losing track of the hospital's objectives. The leaders focused on small and manageable steps for improvement within the PW modules, carefully assessing any resistance to PW from the team and giving constant attention to ensuring the changes took place.

Finally, Lean leaders took time to listen, share information and appreciate the efforts of others. They engaged in individual conversations with nurses in order to stay involved and, if there were issues within the team, to better understand why these had emerged. Information about PW's progress was also shared actively with the team using score charts, newsletters and e-mails: *"It is my responsibility to inform them properly"* (K-01). Lean leaders also expressed appreciation to their team. Compliments were given if the team was on schedule and when certain modules had been completed successfully.

Cross-case analysis

Figure 2 displays the levels of Lean maturity and second-order problem solving of each team. The blue lines represent the assessed levels for each team based on the interviews, while the red line shows the expected level of second-order problem solving, based on the proposition that higher Lean maturity results in increased second-order problem solving.

The graph shows that this proposition did not hold for all the teams. In searching for patterns and reasons, cases that agreed with the proposition were compared to cases that deviated. This analysed whether the difference between actual and expected second-order problem-solving levels could be attributed to the practices of Lean leaders. More specifically, teams C and D (medium Lean maturity – low second-order problem solving i.e. 'ML') were compared with cases E, G, J, M (medium Lean maturity – medium second-order problem solving, i.e. 'MM'). Further, cases B and H (high Lean maturity – medium second-order problem solving, i.e. 'HM') were compared with team A (high Lean maturity – high second-order problem solving, i.e. 'HH').

Please insert figure 2 here

For peer review only - http://bmjopen.bmj.com/site/about/guidelines.xhtml

Our analysis shows that Lean leaders are able to contribute to their team achieving a high level of second-order problem solving. Second-order problem solving can be increased by leadership practices that encourage team ownership in the problem-solving process. In the HH case, team A, the leader played a crucial role in empowering and stimulating nurses to take the lead in thinking about solutions to problems and in implementing solutions through a bottom-up approach. This was explained as follows: *"She [the team leader] tells me to go and investigate how I could make a solution happen"* (A-03). In comparison, in the HM cases, the teams were self-managed to some extent, but responsibility over the entire problem-solving process had not been completely relinquished by the leaders. That is, in the latter, more involvement and active support of the leaders in the problem-solving process was observed.

Second, our analysis shows that an enthusiastic leader who actively participates in the Lean initiative as a role model can lead to a higher level of second-order problem solving. In the HH and MM teams, the leaders were seen as role models for PW through their enthusiasm and participation, as exemplified by the following quote: *"Our leader is very enthusiastic and fanatical"* (E-02). This was perceived as an important aspect that keeps PW alive in the team and, thereby, keeps nurses motivated to participate. In contrast, in the HM and ML cases, the level of enthusiasm and participation from leaders was perceived as being lower.

Discussion

The first main research question posed in this research was: *How does Lean maturity influence the problem-solving behaviour of nursing teams?* Our findings confirm the related proposition that, as nursing teams reach higher levels of Lean maturity, they also demonstrate higher degrees of second-order problem solving. As such, we add to the evidence for the existence of a positive relationship between Lean maturity and second-order problem solving.[5] This suggests that nurses may become more skilled in discovering and removing the root causes of organizational problems through Lean adoption.[19, 20] The reason is that Lean helps nurses to become acquainted with and involved in the identification, analysis and removal of root causes of problems. In the PW programme, nursing teams were trained to use the Hairdryer model that involves multiple activities associated with second-order problem-solving actions.[8]

The second research question was: *What constitutes Lean leadership in nursing wards?* In order to answer this question, Lean leadership was studied empirically in several nursing teams, resulting in the identification of seven Lean leadership practices as listed in the within-case analysis section above. Overall, our findings indicate that Lean leadership in nursing wards has strong connections with transformational leadership theory, a finding in line with earlier studies.[11, 14]

That is, many of the identified Lean leadership practices can be classified as transformational. For example, arousing the team spirit through motivation and inspiration are key activities in the inspirational motivation dimension.[15] Also, charismatic-inspirational leadership was in evidence when Lean leaders enthused others and, at the same time, actively participated in the Lean programme themselves.[21] A further connection with individualized consideration and coaching to develop followers was observed in the Lean leaders' practice of unlocking individual and team potential. This was achieved by recognizing differences in individuals' qualities and preferences within the team.[21] Our results indicate that Lean leaders also used other transformational practices, such as listening effectively, actively sharing information and appreciating people.[21] Our results also underpin the importance of visualization

For peer review only - http://bmjopen.bmj.com/site/about/guidelines.xhtml

in applying Lean in hospital environments.[22] Visualization was practiced by Lean leaders to help their team identify areas for improvement[11] and to demonstrate PW accomplishments on improvement boards that were located in the nursing wards.

The final research question was: *How does Lean leadership affect the relationship between Lean maturity and second-order problem solving?* The cross-case analysis revealed that successful Lean leaders increasingly relinquished responsibility for improvement activities to their team as Lean maturity increased. At the outset of Lean initiatives, large investments from leaders were required in terms of effort and resources[11] and top-down steering may initially be necessary to create the supporting structure for Lean.[23] However, in later stages, it becomes imperative to empower frontline staff so that they can take the initiative in daily improvement activities.[12] Our findings suggest that such a transition was necessary for nursing teams to reach a high level of second-order problem solving. Lean leaders have an important role in facilitating this transition by encouraging nurses to take ownership of improvement activities and by developing their team to become self-managing. The importance of stimulating a bottom-up approach to continuous improvement in healthcare is supported by existing literature.[20, 22]

Overall, our findings suggest that successful Lean implementation in nursing teams requires a bottom-up approach with responsibility for improvement activities gradually being handed over to frontline nurses, supported by leaders with strong transformational leadership skills that radiate enthusiasm towards their team. Through this, nursing teams can achieve a high second-order problem-solving level as Lean matures.

This study has a number of limitations. First, since nursing teams from only one Dutch hospital were included, the external validity of our findings could be impacted negatively. However, this choice had the benefit that it provided nursing teams with a range of Lean maturities working under similar circumstances. Second, when we started the interview round, we realized that many respondents did not recognize all the problems provided in the scenarios of Gemmel et al.[5] This was counteracted at an early stage of data collection by asking an additional open-ended question as to whether nurses could provide an example of a problem solved through a second-order problem-solving approach. This resulted in many useful responses.

Further research could aim to improve the instrument for measuring second-order problem solving in nursing teams. This could involve adding new scenarios in which nurses are able to recognize their problem-solving behaviour. Other research that wants to measure Lean maturity may benefit from adopting the validated scale, involving six items from the Malmbrandt and Ahlström's more detailed instrument[1], that this study found to be relevant and measurable in nursing wards.

Acknowledgement
We would like to thank Iris Brouwer, Pauline Vinks and Thomas Fransen for their contributions to the design, data collection and data analysis of the study. We are further grateful to the study participants from the hospital.

Funding
No funding has been received for this research.

Competing interests
The authors declare that they have no competing interests.

Contributions

For peer review only - http://bmjopen.bmj.com/site/about/guidelines.xhtml

AB contributed to the conception and design of the study, data analysis, interpretation of the data and in drafting the manuscript. AB and KA analysed the Lean leadership interview data. KA and GR contributed to the study's conception and design, and to revising the paper. PG and BM shared the scenarios from a prior study and contributed to revising the paper. KA coordinated the study. Finally, all authors read and approved the submitted manuscript. SQUIRE guidelines were used to check the completeness of the reporting.[24]

Data sharing statement
No additional data are available.

Ethical approval
The Institutional Review Board (IRB) of the Faculty of Economics and Business has reviewed the Research Data Management Plan/Ethics checklist and came to the conclusion that 'the study entails no risk for participants, that anonymity is assured, that participants are not misled and that they are informed about their participation and have the choice to decline such participation' (reference #RDMPFEB-20180723-7173). All participants in the study gave informed consent before taking part. For this research, no approval of a medical ethical committee was required according to the criteria of the Dutch law.

References
1. Malmbrandt M, Åhlström P. An instrument for assessing lean service adoption. *Int J Oper Prod Man.* 2013;33(9):1131-65.
2. Gioia DA, Corley KG, Hamilton AL. Seeking qualitative rigor in inductive research notes on the Gioia methodology. *Organ Res Methods.* 2013;16(1):15-31.
3. Mazzocato P, Holden RJ, Brommels, M, *et al*. How does lean work in emergency care? A case study of a lean-inspired intervention at the Astrid Lindgren Children's hospital, Stockholm, Sweden. *BMC Health Serv Res*. 2012;12;28.
4. Kaltenbrunner M, Bengtsson L, Mathiassen SE, *et al*. A questionnaire measuring staff perceptions of Lean adoption in healthcare: development and psychometric testing. *BMC Health Serv Res*. 2017;17;235.
5. Gemmel P, Van Beveren S, Landry S, *et al*. Problem-solving behavior of nurses in a lean environment. *J Nurs Manage*. 2018(f.c.).
6. Burgess N, Radnor Z. Service improvement in the English National health service: complexities and tensions. *J Manage Organ.* 2012;18(5):594-607.
7. Aij KH, Visse M, Widdershoven GA. Lean leadership: an ethnographic study. *Leadersh Health Serv.* 2015;28(2):119-34.
8. Tucker AL, Edmondson AC. Why hospitals don't learn from failures: organizational and psychological dynamics that inhibit system change. *Calif Manage Rev.* 2003;45(2):55-72.
9. Mazur LM, Chen SG. An empirical study for medication delivery improvement based on healthcare professionals' perceptions of medication delivery system. *Health Care Manag Sci.* 2009;12(1):56-66.
10. Aij KH, Teunissen M. Lean leadership attributes: a systematic review of the literature. *J Health Organ Manag*. 2017;31(7/8):713-29.
11. Poksinska B, Swartling D, Drotz E. The daily work of Lean leaders–lessons from manufacturing and healthcare, *Total Qual Manag Bus.* 2013;24(7-8):886-98.
12. Goodridge D, Westhorp G, Rotter T, *et al*. Lean and leadership practices: development of an initial realist program theory. *BMC Health Serv Res.* 2015;15;362.
13. Tucker AL. Workarounds and resiliency on the front lines of health care. 2009. Available from https://www.ahrq.gov/ (patient safety network AHRQ).
14. Van Rossum L, Aij KH, Simons FE, *et al*. Lean healthcare from a change management perspective: the role of leadership and workforce flexibility in an operating theatre. *J Health Organ Manag.* 2016;30(3):475-93.
15. Bass BM. Two decades of research and development in transformational leadership. *Eur J Work Organ Psy*. 1999;8(1):9-32.

For peer review only - http://bmjopen.bmj.com/site/about/guidelines.xhtml

16. White M, Wells JS, Butterworth T. The impact of a large-scale quality improvement programme on work engagement: Preliminary results from a national cross-sectional-survey of the 'Productive Ward'. *Int J Nurs Stud.* 2014;51(12):1634-43.
17. Karlsson C. *Researching operations management.* New York: Routledge, 2009.
18. Krippendorff K. Reliability in content analysis. *Hum Commun Res*. 2004;30:411-33.
19. Mazur LM, Chen SG, Prescott B. Pragmatic evaluation of the Toyota Production System (TPS) analysis procedure for problem solving with entry-level nurses. *J Ind Eng Manag.* 2008;1(2):240-68.
20. Spear SJ. Fixing health care from the inside, today. *Harvard Bus Rev*. 2005;83(9):78-91.
21. Stone AG, Russell RF, Patterson K. Transformational versus servant leadership: A difference in leader focus. *Leadership Org Dev J.* 2004;25(4):349-61.
22. Drotz E, Poksinska B. Lean in healthcare from employees' perspectives. *J Health Organ Manag*. 2014;28(2):177-95.
23. Taher D, Landry S, Toussaint J. Breadth vs. depth: How to start deploying the daily management system for your lean transformation. *J Hosp Adm.* 2016;5(6):90-6.
24. Ogrinc G, Davies L, Goodman D, *et al*. SQUIRE 2.0 (Standards for QUality Improvement Reporting Excellence): revised publication guidelines from a detailed consensus process. *BMJ Qual Saf.* 2015;0:1-7.

Figure Legends:

Figure 1 Conceptual model

Figure 2 Lean maturity levels & expected (dotted in red) and actual (blue) second-order problem-solving level per case/team

For peer review only

For peer review only - http://bmjopen.bmj.com/site/about/guidelines.xhtml

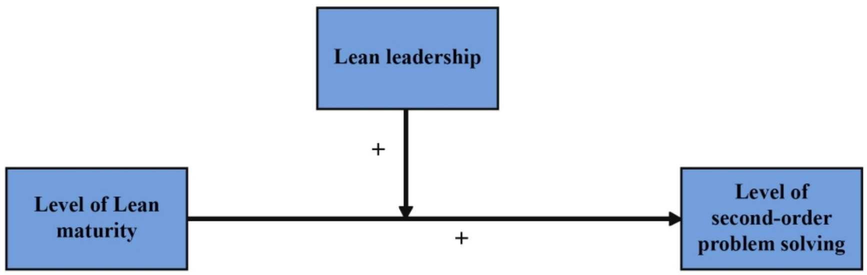

Figure 1 Conceptual model

Figure 1 Conceptual model

750x416mm (72 x 72 DPI)

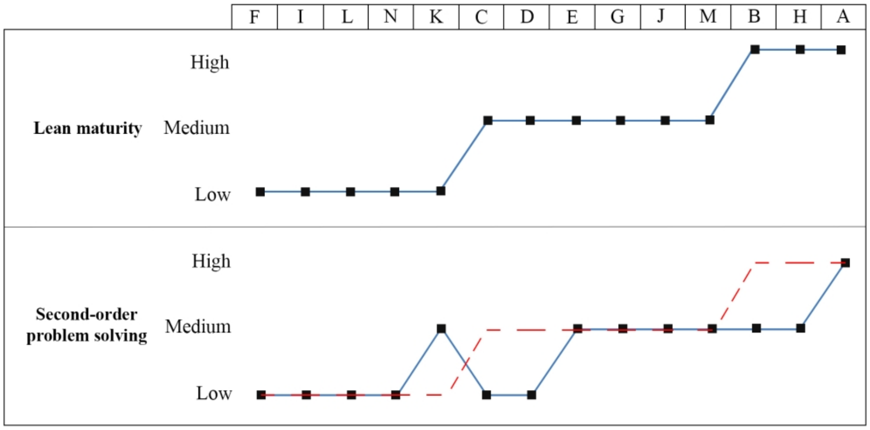

Figure 2 Lean maturity levels & expected (dotted in red) and actual (blue) second-order problem-solving level per case/team

Figure 2 Lean maturity levels & expected (dotted in red) and actual (blue) second-order problem-solving level per case/team

750x416mm (72 x 72 DPI)

Reporting checklist for quality improvement study.

Based on the SQUIRE guidelines.

Instructions to authors

Complete this checklist by entering the page numbers from your manuscript where readers will find each of the items listed below.

Your article may not currently address all the items on the checklist. Please modify your text to include the missing information. If you are certain that an item does not apply, please write "n/a" and provide a short explanation.

Upload your completed checklist as an extra file when you submit to a journal.

		Reporting Item	Page Number
	#1	Indicate that the manuscript concerns an initiative to improve healthcare (broadly defined to include the quality, safety, effectiveness, patientcenteredness, timeliness, cost, efficiency, and equity of healthcare)	1, 4
	#02a	Provide adequate information to aid in searching and indexing	1
	#02b	Summarize all key information from various sections of the text using the abstract format of the intended publication or a structured summary such as: background, local problem, methods, interventions, results, conclusions	1
Problem description	#3	Nature and significance of the local problem	2
Available knowledge	#4	Summary of what is currently known about the problem, including relevant previous studies	2, 3
Rationale	#5	Informal or formal frameworks, models, concepts, and / or theories used to explain the problem, any reasons or assumptions that were used to develop the intervention(s), and reasons why the intervention(s) was expected to work	4
Specific aims	#6	Purpose of the project and of this report	2

For peer review only

		Reporting Item	Page Number
Context	#7	Contextual elements considered important at the outset of introducing the intervention(s)	4
Intervention(s)	#08a	Description of the intervention(s) in sufficient detail that others could reproduce it	4
	#08b	Specifics of the team involved in the work	4
Study of the Intervention(s)	#09a	Approach chosen for assessing the impact of the intervention(s)	4, 5
	#09b	Approach used to establish whether the observed outcomes were due to the intervention(s)	4, 5
Measures	#10a	Measures chosen for studying processes and outcomes of the intervention(s), including rationale for choosing them, their operational definitions, and their validity and reliability	4, 5
	#10b	Description of the approach to the ongoing assessment of contextual elements that contributed to the success, failure, efficiency, and cost	4, 5
	#10c	Methods employed for assessing completeness and accuracy of data	4, 5
Analysis	#11a	Qualitative and quantitative methods used to draw inferences from the data	4, 5
	#11b	Methods for understanding variation within the data, including the effects of time as a variable	5
Ethical considerations	#12	Ethical aspects of implementing and studying the intervention(s) and how they were addressed, including, but not limited to, formal ethics review and potential conflict(s) of interest	9, 10
	#13a	Initial steps of the intervention(s) and their evolution over time (e.g., time-line diagram, flow chart, or table), including modifications made to the intervention during the project	5, 6, 7
	#13b	Details of the process measures and outcome	5, 6, 7
	#13c	Contextual elements that interacted with the intervention(s)	6, 7
	#13d	Observed associations between outcomes, interventions, and relevant contextual elements	5, 6, 7

For peer review only

		Reporting Item	Page Number
	#13e	Unintended consequences such as unexpected benefits, problems, failures, or costs associated with the intervention(s).	n/a
	#13f	Details about missing data	n/a
Summary	#14a	Key findings, including relevance to the rationale and specific aims	8, 9
	#14b	Particular strengths of the project	1
Interpretation	#15a	Nature of the association between the intervention(s) and the outcomes	8, 9
	#15b	Comparison of results with findings from other publications	8, 9
	#15c	Impact of the project on people and systems	n/a
	#15d	Reasons for any differences between observed and anticipated outcomes, including the influence of context	8, 9
	#15e	Costs and strategic trade-offs, including opportunity costs	n/a
Limitations	#16a	Limits to the generalizability of the work	2, 9
	#16b	Factors that might have limited internal validity such as confounding, bias, or imprecision in the design, methods, measurement, or analysis	9
	#16c	Efforts made to minimize and adjust for limitations	9
Conclusion	#17a	Usefulness of the work	1, 8
	#17b	Sustainability	n/a
	#17c	Potential for spread to other contexts	9
	#17d	Implications for practice and for further study in the field	9
	#17e	Suggested next steps	9
Funding	#18	Sources of funding that supported this work. Role, if any, of the funding organization in the design, implementation, interpretation, and reporting	n/a

The SQUIRE 2.0 checklist is distributed under the terms of the Creative Commons Attribution License CC BY-NC 4.0. This checklist was completed on 17. September 2018 using http://www.goodreports.org/, a tool made by the EQUATOR Network in collaboration with Penelope.ai

For peer review only

BMJ Open

The role of lean leadership in the lean maturity – second-order problem-solving relationship: a mixed methods study

Journal:	*BMJ Open*
Manuscript ID	bmjopen-2018-026737.R1
Article Type:	Research
Date Submitted by the Author:	31-Jan-2019

Complete List of Authors:	Bijl, Arie; University of Groningen, Faculty of Economics and Business, Department of Operations,<br>Ahaus, Kees; University of Groningen, Faculty of Economics and Business, Department of Operations,<br>Ruël, Gwenny; University of Groningen, Faculty of Economics and Business, Department of Operations,<br>Gemmel, Paul; Ghent University, Faculty of Economics and Business Administration, Department of Innovation, Entrepreneurship and Service Management,<br>Meijboom, Bert; Tilburg University, Department of Tranzo

**Primary Subject Heading**:	Health services research
Secondary Subject Heading:	Health services research
Keywords:	Lean leadership, Lean maturity, second-order problem solving

SCHOLARONE™
Manuscripts

The role of lean leadership in the lean maturity – second-order problem-solving relationship: a mixed methods study

Arie Bijl
Department of Operations
Faculty of Economics and Business
University of Groningen, The Netherlands
Nettelbosje 2
9747 AE Groningen, The Netherlands
T. +31503637078
Email address: ariebijl94@gmail.com

Kees Ahaus **(corresponding author)**
Department of Operations
Faculty of Economics and Business
University of Groningen, The Netherlands
Nettelbosje 2
9747 AE Groningen, The Netherlands
T. +31503637078, M. +31651591859
Email address: c.t.b.ahaus@rug.nl

Gwenny Ruël
Department of Operations
Faculty of Economics and Business
University of Groningen, The Netherlands
Nettelbosje 2
9747 AE Groningen, The Netherlands
T. +31503637078
Email address: g.c.ruel@rug.nl

Paul Gemmel
Department of Innovation, Entrepreneurship and Service Management
Faculty of Economics and Business Administration
Ghent University, Belgium
Email address: Paul.Gemmel@ugent.be
Tweekerkenstraat 2
9000 Gent, Belgium
T. +3292643518

Bert Meijboom
Department of Tranzo
Tilburg University, the Netherlands
Email address: B.R.Meijboom@uvt.nl
Postbus 90153
5000 LE Tilburg, The Netherlands
T. +31134663237

For peer review only

The role of lean leadership in the lean maturity – second-order problem-solving relationship: a mixed methods study

ABSTRACT

Objectives: To investigate the relationship between Lean adoption and problem-solving behaviour in nursing teams, and to explore the practices of Lean leaders in nursing wards to reveal how they can stimulate second-order problem solving within their teams.

Design: A mixed-methods retrospective multiple case study using semi-structured interviews. Interview data were used to assess the level of Lean maturity (based on a customized validated instrument) and the level of second-order problem solving (based on scenarios). Within-case and cross-case analyses were employed to identify Lean leadership practices.

Setting: 14 nursing teams, with different levels of Lean maturity, in a Dutch hospital.

Participants: Three members of each nursing team were interviewed: the team leader, one nurse from the ward's core team for the Lean-based quality improvement programme and one nurse outside the core team.

Interventions: The nursing teams were in various phases of a Lean-based quality improvement programme: 'The Productive Ward – Releasing Time to Care'.

Results: A strongly significant positive relationship between Lean maturity and second-order problem solving was found: $\beta = 0.68$, $R^2 = 0.46$, $p < 0.001$. Further, the results indicated a potential strengthening effect of Lean leadership on this relationship. Seven Lean leadership practices emerged from the data collected in a nursing ward setting: (1) convincing and setting an example; (2) unlocking individual and team potential; (3) solving problems systematically; (4) enthusing, actively participating and visualizing; (5) developing self-managing teams; (6) sensing, as orchestrator, what is needed for change; and (7) listening, sharing information and appreciating. These practices have a strong link with transformational leadership.

Conclusions: As Lean matures, nursing teams reach a higher level of second-order problem solving. In later stages, Lean leaders increasingly relinquish responsibility by developing self-managing teams.

Keywords: Lean leadership, Lean maturity, second-order problem solving

Word Count: 4,407

Strengths and limitations of this study

- The study fills a gap in knowledge as to whether more-mature Lean teams show a higher level of second-order problem-solving behaviour.
- In addition, it provides valuable insight into typical Lean leadership practices and how these are linked to mainstream leadership theories.
- Future research attempting to measure Lean maturity in healthcare environments may benefit from using the newly developed and validated scale that incorporates six items from Malmbrandt and Ahlström's instrument.
- Another strength of the study is its use of Gioia methodology to inductively analyse the Lean leadership data.

For peer review only - http://bmjopen.bmj.com/site/about/guidelines.xhtml

For peer review only

- A limitation is that the nursing teams all came from one Dutch hospital, however, this choice had the benefit that it provided nursing teams with a range of Lean maturities working under similar circumstances.

Introduction

The earliest applications of Lean thinking in the healthcare sector were more than a decade ago. Since then, Lean has gained in popularity in the sector.[1] The primary goals of Lean in healthcare have been to increase the quality of care and to increase efficiency.[2] To achieve these goals, most healthcare organizations have emphasized the application of Lean tools to reduce direct waste, but neglected developing the problem-solving abilities of frontline employees.[3] This approach may have created some process improvements, but long-term hospital-wide benefits have rarely been achieved.[4]

To realize more of Lean's potential, it is often suggested that structured problem solving should be developed throughout the organization to sustainably improve processes.[3] This is inspired by Liker's 4P model that identifies four aspects of Lean: philosophy, process, people and problem-solving.[3] One well-known and effective approach to this is *second-order problem solving*. This involves the in-depth questioning of work practices to uncover and remove the root causes of problems.[5] This approach is in sharp contrast with *first-order problem solving* where problems are resolved in an ad-hoc manner, while underlying causes remain.[6] A recent study by Gemmel *et al.*[3] suggests that second-order problem solving is more prevalent than first-order problem solving in nursing teams with high levels of Lean maturity. However, evidence from a larger sample of wards is needed to confirm this.

Earlier studies have suggested that team leaders could stimulate second-order problem-solving behaviour by nurses.[5] Team leaders are crucial in creating and sustaining the benefits of Lean adoption in hospital wards since they can help to create a culture of continuous improvement, empower frontline employees and foster participation.[7, 8, 9] Although the literature often emphasizes the importance of Lean leadership, there are few empirical studies of Lean leadership in healthcare.[9, 10] Moreover, previous studies have not connected Lean leadership with other leadership theories.[9] Consequently, an in-depth empirical study of Lean leadership in nursing wards is needed, whereby the identified leadership practices can be positioned within existing leadership approaches in order to enhance our understanding of this concept.

In this paper, we study the relationship between Lean maturity and second-order problem-solving behaviour. In addition, we explore the meaning attached to Lean leadership in nursing wards and discover how Lean leadership moderates the Lean maturity – second-order problem-solving relationship. In our study of the Lean leadership concept, we focus solely on identifying leadership practices in nursing wards (i.e. in terms of team leadership). We answer the following research questions:

- How does Lean maturity affect the problem-solving behaviour of nursing teams?
- What constitutes Lean leadership in nursing wards?
- How does Lean leadership affect the relationship between Lean maturity and second-order problem solving?

Background

Lean maturity

The concept of Lean maturity needs careful consideration if one is to accurately assess the impact of Lean implementation on the problem-solving behaviour of nursing teams.[3] Malmbrandt and Åhlström[11] argue that the extent of an organization's Lean

For peer review only - http://bmjopen.bmj.com/site/about/guidelines.xhtml

adoption can be measured using an instrument that incorporates measures to assess Lean enablers, Lean practices and performance. Lean enablers represent the supporting structure or preconditions of Lean, including the training of employees and dedicating time and resources to improvement work. Lean practices correspond to Lean principles, such as continuous improvement and eliminating waste. Performance refers to the results of Lean adoption in terms of measures such as quality, customer satisfaction and costs. Together, these three dimensions determine the Lean maturity level of an organization. These can range from no adoption through the increase of continuous improvement activities to an exceptional, well-defined and innovative approach.[11]

Second-order problem-solving behaviour

Nurses, as frontline service providers, play an essential role and are in the best position to uncover and eliminate the root causes of problems and, thereby, help their team learn.[5, 6] Second-order problem solving occurs when "the worker, in addition to patching the problem so that the immediate task at hand can be completed (i.e. first-order problem solving), also takes action to address underlying causes"[6(p.63)].

Tucker and Edmondson[5(p.61)] distinguish five broad actions linked to second-order problem solving: (1) communicating to the person or department responsible for the problem; (2) bringing the problem to the manager's attention; (3) sharing ideas about the cause of the situation and how to prevent recurrence; (4) implementing changes; and (5) verifying that changes have the desired effect.

Nurses apply both first- and second-order problem-solving approaches. Quick workarounds may be needed if patients cannot wait for the promised care.[12] However, only by applying second-order problem solving can real process improvements be made. Gemmel *et al.*[3] go as far as to state that a learning organization based on second-order problem solving should be one of the ultimate goals of Lean.

Lean leadership

The importance of effective 'Lean leadership' during Lean implementations is widely recognized.[8] A few studies in healthcare have suggested that Lean leadership can be linked to transformational leadership theory.[9, 13] Under transformational leadership, followers are motivated to do more than originally expected and to feel trust, loyalty, respect and admiration towards their leader.[14] Transformational leaders develop their followers through four key dimensions. *Idealized influence* refers to a leader being a role model, one who is admired and respected by their followers. *Inspirational motivation* implies that a leader motivates those around him/her and arouses their spirit. Transformational leaders use *intellectual stimulation* to stimulate their followers to be innovative and creative. Finally, a transformational leader attends to the need for achievement and growth of individuals through *individual consideration* by acting as a coach.[14] Transformational leadership is different from transactional leadership. The latter is merely an exchange process to motivate follower compliance, where a leader clarifies performance criteria, states expectations and determines what followers receive in return.[14] The link between transformational leadership theory and Lean leadership is relatively under-researched.[9] In healthcare, there are only a few empirical studies investigating Lean leadership practices.[9, 10]

Conceptual model

Figure 1 displays the conceptual model applied in this study. First, in line with Gemmel *et al.*[3], we expect greater Lean maturity to potentially lead to an elevated degree of

For peer review only - http://bmjopen.bmj.com/site/about/guidelines.xhtml

second-order problem solving in nursing teams. Second, since Lean leaders are seen as essential in enhancing the problem-solving abilities of healthcare staff[8], we expect effective Lean leadership to strengthen the relationship between Lean maturity and second-order problem solving.

Please insert figure 1 here

Methods

This research involves 14 case studies of nursing teams in different departments of a Dutch hospital, all in different phases of a Lean-based quality improvement programme: 'The Productive Ward – Releasing Time to Care' (from now on referred to as PW). This programme trains nursing teams in how to apply Lean tools and principles in their daily work and claims to increase the time nurses have for direct patient care.[15] A key tool incorporated in PW is the Hairdryer Model. This teaches nurses how to engage in effective problem solving by discussing problems in groups, mapping the current situation, collecting data, implementing changes and assessing their impacts.

Each of the 14 nursing teams has a PW core team that leads the change. Generally, this consists of the team leader and a few nurses. To obtain reliable data, the team leader, one nurse inside and one nurse outside each PW core team were interviewed. The sample consisted of 7 males and 35 females; 14 of whom were employed for less than five years, 15 between five and ten years, 5 between ten and fifteen years, 1 between fifteen and twenty years and 7 who had worked twenty or more years in the hospital. The nursing teams came from various wards: cardiology, urology, orthopaedics, CCU, emergency department, acute admission, neurology, lung medicine, birth centre, neonatology, paediatrics, oncology, short-stay, and day nursing. Nursing teams with differing periods in the programme (0-24 months) were selected as cases. We expected this to provide us with variation on the Lean maturity variable. Cases were selected with different durations in the belief this would produce different results but for predictable reasons (enabling theoretical replication).[16] Table 1 shows the selected cases within each duration period.

Table 1 Selected cases within each duration period

**Duration**	Selected cases
**Period 1 (0-6 months)**	L, N
**Period 2 (6-12 months)**	H, I, J, K
**Period 3 (12-18 months)**	D, E, F, G, M
**Period 4 (18-24 months)**	B, C
**Period 5 (24 + months)**	A

Data collection

In total, 42 semi-structured interviews with nurses were conducted in December 2016 with an average duration of 40 minutes (minimum 24 minutes, maximum 68 minutes). More than one researcher was present at each interview. The interview questions related to Lean maturity were designed to measure several Lean enablers and Lean practices. These provided accurate insight into the progress being made with Lean implementation. These Lean enablers and practices were drawn from Malmbrandt and Åhlström's instrument.[11] Their instrument contains 34 items to assess Lean service adoption but we decided to include only six items for several reasons. First, some items had strong links with our other main variables, such as management commitment and

For peer review only - http://bmjopen.bmj.com/site/about/guidelines.xhtml

understanding (Lean leadership), the degree of structured problem solving (second-order problem solving) and performance and these were excluded to avoid confounding issues between the different concepts/variables of our conceptual model. Secondly, some items were not considered relevant in the hospital context, or too complex to measure (such as the levelling and balancing of workloads). These exclusions resulted in only six items being considered useful for this study. The customized instrument included three Lean enablers (employees' understanding of Lean; time and resources allocated to improvement work; and bi-directional vertical information flows), and three Lean practices (identification of patient value; workplace design for flow; and visualization of information and improvements).

Second-order problem solving was measured through a set of questions in the form of scenarios derived by Gemmel *et al.*[3] These scenarios describe several types of problems nurses face in their daily work. They were used to obtain information about the actions nurses took when faced with a problem. To obtain further insights into this concept, nurses were also asked to provide examples of a problem where they engaged in second-order problem solving. Since the main goal of PW is to increase the proportion of time that nurses spend on direct patient care[15], the level of performance is measured by an open question asking about perceptions of PW outcomes in terms of the time that is freed up for direct patient care.

Lean leadership was studied from various viewpoints, with different sets of open and semi-structured interview questions posed to team leaders and nurses. We specifically asked about leadership practices adopted to support and implement the PW programme and to develop the team in applying PW. Questions related to generic leadership practices were avoided.

Data analysis

From each interview, scores were deduced for the levels of Lean maturity and second-order problem-solving behaviour based on the instruments applied. The scores for each set of three ward members were checked by the interviewers for consistency. This process eventually resulted, for each team, in a low, medium or high rating, for its Lean maturity and its second-order problem-solving level.

As an indicator of the construct validity of our instrument, a correlation analysis was performed to explore whether the duration of the PW programme was related to the level of Lean maturity. This revealed a strong correlation ($r = 0.58$; $p < 0.001$). Next, scores were attributed to the three enablers and three practices of Lean on a five-point scale based on the original instrument by Malmbrandt and Åhlström.[11] Although the Lean enablers and practices were extracted from a validated instrument, a confirmatory factor analysis (CFA) was performed to test the unidimensionality of the six selected items. Since we used an adapted instrument and our sample size was small (N=42) we first looked whether the data fitted a CFA. The KMO = .76 and Bartlett's chi-square = 92.2; $p < .001$ with all communalities > .49 and explaining 56.2% of the variance, which allowed for a CFA. The CFA extracted a single component with all factor loadings above 0.7, indicating the construct validity of measuring Lean maturity using our six-item scale. In addition, the reliability analysis returned a Cronbach's alpha of 0.84 suggesting a strong internal consistency among the Lean enablers and practices.

The information collected through the scenarios was used to link each respondent to a category of first- or second-order problem-solving behaviour. For this, we used a scale with eight levels. Levels 1, 2 and 3 represent first-order problem-solving approaches, whereas levels 4 through 8 represent second-order problem-solving actions as described

For peer review only - http://bmjopen.bmj.com/site/about/guidelines.xhtml

by Tucker and Edmondson[5(p.61)], with level 8 indicating the highest degree of second-order problem solving.

Since second-order problem solving is expected to enhance performance, a correlation analysis was performed to assess this relationship. The perceived performance was categorized using a four-level: (1) perception that there are no clear effects of PW; (2) perception that time is freed up for direct patient care, but only indirectly through a better organized ward; (3) perception that actual time is freed up for direct patient care and this is exemplified; and (4) time is demonstrably freed up for direct patient care.

For the within-case analysis of Lean leadership, data were extracted from all the interviews. Using the rigorous step-by-step approach proposed by Gioia *et al.*[17], the data were coded inductively by two researchers. This led to a coding tree of Lean leadership practices (available on request) involving 339 *in vivo* codes, 29 themes and 7 aggregate dimensions. The list of themes was then used by two researchers to independently code 9 out of 42 interviews (20% of the interviews). A comparison demonstrated an acceptably high inter-rater reliability in terms of assessing Lean leadership with a Krippendorff's alpha value of 0.85.[18]

In the cross-case analysis, cases where consistent low, medium or high levels of both Lean maturity and second-order problem solving were found were compared with cases that deviated from this expectation of consistency to determine whether Lean leadership strengthened the relationship between Lean maturity and second-order problem solving.

Patient and public involvement

There was no patient or public involvement in this study. The results of this work were disseminated to representatives of the hospital. Once the study has been published, the main findings will be used in the education of Master students.

Results

The results showed that increased Lean maturity positively influences the level of second-order problem solving in nursing teams. This relationship was tested through a single linear regression analysis. A strongly significant positive relationship was found between Lean maturity and second-order problem solving: $\beta = 0.68$, $R^2 = 0.46$, $p < 0.001$. There was also a strong correlation between second-order problem solving and performance ($r=0.66$; $p<.001$), indicating that second-order problem solving indeed seems to free up time for patient care.

Within-case analysis

The in-depth analysis of the meaning of Lean leadership resulted in the identification of seven Lean leadership practices: (1) convincing and setting an example; (2) unlocking individual and team potential; (3) solving problems systematically; (4) enthusing, actively participating and visualizing; (5) developing self-managing teams; (6) sensing, as orchestrator, what is needed for change; and (7) listening, sharing information and appreciating. Each of these practices is explained below.

First, Lean leaders helped nurses to become acquainted with the programme. They constitute the driving force of PW by setting an example and actively promoting PW in their team. Initially, many nurses needed to be convinced of the value of PW and, so, an important activity was to explain PW modules and highlight potential benefits. These included the time that could be saved by applying PW: *"I try to give insights into why we have to change certain things"* (D-01: in this notation, D refers to team D,

For peer review only - http://bmjopen.bmj.com/site/about/guidelines.xhtml

participant ‘01’ refers to the team leader; ‘02’ and ‘03’ refer to the nurses inside and outside the PW core team respectively).

Lean leaders also aimed to unlock the greater potential of nurses and their team by encouraging everyone to become active within PW, for instance, by stimulating membership of the PW core team and participation in improvement board sessions. Nurses were also invited to indicate a preferred improvement project, based on their own interests and capabilities, to work on within PW. Within these PW projects, Lean leaders adjusted their own degree of involvement for each individual based on the level of experience of each nurse. This gave room for individual potential to develop.

Further, Lean leaders were involved in systematic problem solving. They were present during team discussions where problems were analysed by applying PW tools. Leaders also encouraged their team to solve problems through the Hairdryer Model, as they felt that this model could be incorporated in nurses’ work practices. In addition, the leaders cooperated with nurses who wanted to engage in systematic problem solving, as one of the participants stated: *“Our team leader would help us by providing input on how we can approach things”* (C-03).

Another key Lean leadership practice was to enthuse the team, actively participate in PW and visualize. First, the leaders encouraged nurses to organize improvement board sessions and to carry out daily evaluations of processes. We observed that core-team members were regularly rotated, as being a member of the PW core team was perceived as a strong motivating factor by nurses. This leadership involvement was seen as highly important by both nurses and leaders, as the following quotes illustrate: *“The leader motivates me through her enthusiasm, and by showing that she wants it herself”* (N-02) and: *“I need to bring along the group through enthusiasm”* (E-01). Finally, many participants pointed out that leaders used visualization through photographs or videos to create awareness of a non-optimal situation and to demonstrate PW accomplishments.

Lean leaders also developed self-managing teams. They encouraged nurses to actively take the lead, for instance, by giving nurses ownership of improving certain processes through PW tools. It was frequently noted that, as the implementation of PW progressed, Lean leaders would increasingly relinquish tasks and responsibility to increase the sense of ownership among the nurses in the team. This interpretation is supported by the following quotes: *“Through this programme, my tasks are diminished as nurses themselves take more responsibility”* (A-01) and *“The leader does not want to keep tight control of everything, he relinquishes tasks and gives guidance, which motivates us to be actively involved in the programme”* (H-03).

Lean leaders continuously thought about what was needed for change to take place. For instance, they facilitated this by providing time and resources to engage in PW activities and they ensured that PW meetings regularly took place. It was also stated that the leaders provided direction and kept an overview of PW’s progress within their team, without losing track of the hospital’s objectives. The leaders focused on small and manageable steps for improvement within the PW modules, carefully assessing any resistance to PW from the team and giving constant attention to ensuring the changes took place.

Finally, Lean leaders took time to listen, share information and appreciate the efforts of others. They engaged in individual conversations with nurses in order to stay involved and, if there were issues within the team, to better understand why these had emerged. Information about PW’s progress was also shared actively with the team using score charts, newsletters and e-mails: *“It is my responsibility to inform them properly”* (K-01). Lean leaders also expressed appreciation to their team. Compliments were given if the team was on schedule and when certain modules had been completed successfully.

For peer review only - http://bmjopen.bmj.com/site/about/guidelines.xhtml

Cross-case analysis

Figure 2 displays the levels of Lean maturity and second-order problem solving of each team. The blue lines represent the assessed levels for each team based on the interviews, while the red line shows the expected level of second-order problem solving, based on the proposition that higher Lean maturity results in increased second-order problem solving.

The graph shows that this proposition did not hold for all the teams. In searching for patterns and reasons, cases that agreed with the proposition were compared to cases that deviated. This analysed whether the difference between actual and expected second-order problem-solving levels could be attributed to the practices of Lean leaders. More specifically, teams C and D (medium Lean maturity – low second-order problem solving i.e. 'ML') were compared with cases E, G, J, M (medium Lean maturity – medium second-order problem solving, i.e. 'MM'). Further, cases B and H (high Lean maturity – medium second-order problem solving, i.e. 'HM') were compared with team A (high Lean maturity – high second-order problem solving, i.e. 'HH').

Please insert figure 2 here

Our analysis shows that Lean leaders are able to contribute to their team achieving a high level of second-order problem solving. Second-order problem solving can be increased by leadership practices that encourage team ownership of the problem-solving process. In the HH case, team A, the leader played a crucial role in empowering and stimulating nurses to take the lead in thinking about solutions to problems and in implementing solutions through a bottom-up approach. This was explained as follows: *"She [the team leader] tells me to go and investigate how I could make a solution happen"* (A-03). In comparison, in the HM cases, the teams were self-managed to some extent, but responsibility for the entire problem-solving process had not been completely relinquished by the leaders. That is, in the latter, more involvement and active support of the leaders in the problem-solving process was observed.

Second, our analysis shows that an enthusiastic leader who actively participates in the Lean initiative as a role model can lead to a higher level of second-order problem solving. In the HH and MM teams, the leaders were seen as role models for PW through their enthusiasm and participation, as exemplified by the following quote: *"Our leader is very enthusiastic and fanatical"* (E-02). This was perceived as an important aspect that keeps PW alive in the team and, thereby, keeps nurses motivated to participate. In contrast, in the HM and ML cases, the level of enthusiasm and participation from leaders was perceived as being lower.

Discussion

The first main research question posed in this research was: *How does Lean maturity influence the problem-solving behaviour of nursing teams?* Our findings confirm the related proposition that, as nursing teams reach higher levels of Lean maturity, they also demonstrate higher degrees of second-order problem solving. As such, we add to the evidence for the existence of a positive relationship between Lean maturity and second-order problem solving.[3] This suggests that nurses may become more skilled in discovering and removing the root causes of organizational problems through Lean adoption.[19, 20] The reason is that Lean helps nurses to become acquainted with and involved in the identification, analysis and removal of the root causes of problems. In the PW programme, nursing teams were trained to use the Hairdryer Model that involves multiple activities associated with second-order problem-solving actions.[5]

For peer review only - http://bmjopen.bmj.com/site/about/guidelines.xhtml

The second research question was: *What constitutes Lean leadership in nursing wards?* In order to answer this question, Lean leadership was studied empirically in several nursing teams, resulting in the identification of seven Lean leadership practices as listed in the within-case analysis section. Overall, our findings indicate that Lean leadership in nursing wards has strong connections with transformational leadership theory, a finding in line with earlier studies.[9, 13] That is, many of the identified Lean leadership practices can be classified as transformational. For example, arousing the team spirit through motivation and inspiration are key activities in the inspirational motivation dimension.[14] Charismatic-inspirational leadership was also in evidence when Lean leaders enthused others and, at the same time, actively participated in the Lean programme themselves.[21] A further connection with individualized consideration and coaching to develop followers was observed in the Lean leaders' practice of unlocking individual and team potential. This was achieved by recognizing differences in individuals' qualities and preferences within the team.[21] Our results indicate that Lean leaders also used other transformational practices, such as listening effectively, actively sharing information and appreciating people.[21] Our results also underpin the importance of visualization in applying Lean in hospital environments.[22] Visualization was practiced by Lean leaders to help their team identify areas for improvement[9] and to demonstrate PW accomplishments on improvement boards that were located in the nursing wards.

The final research question was: *How does Lean leadership affect the relationship between Lean maturity and second-order problem solving?* The cross-case analysis revealed that successful Lean leaders increasingly relinquished responsibility for improvement activities to their team as Lean maturity increased. At the outset of Lean initiatives, large investments from leaders were required in terms of effort and resources[9] and top-down steering may initially be necessary to create the supporting structure for Lean.[23] However, in later stages, it becomes imperative to empower frontline staff so that they can take the initiative in daily improvement activities.[10] Our findings suggest that such a transition was necessary for nursing teams to reach a high level of second-order problem solving. Lean leaders have an important role in facilitating this transition by encouraging nurses to take ownership of improvement activities and by developing their team to become self-managing. The importance of stimulating a bottom-up approach to continuous improvement in healthcare is supported by existing literature.[20, 22]

Overall, our findings suggest that successful Lean implementation in nursing teams requires a bottom-up approach with responsibility for improvement activities gradually being handed over to frontline nurses, supported by leaders with strong transformational leadership skills that radiate enthusiasm towards their team. Through this, nursing teams can achieve a high second-order problem-solving level as Lean matures.

This study has a number of limitations. First, since nursing teams from only one Dutch hospital were included, the external validity of our findings could be impacted negatively. However, this choice had the benefit that it enabled us to compare nursing teams with a range of Lean maturities working under similar circumstances. Second, when we started the interview round, we realized that many respondents did not recognize all the problems provided in the scenarios of Gemmel et al.[3] This was counteracted at an early stage of data collection by asking an additional open-ended question as to whether nurses could provide an example of a problem solved through a second-order problem-solving approach. This resulted in many useful responses.

Further research could aim to improve the instrument for measuring second-order problem solving in nursing teams. This could involve adding new scenarios in which

For peer review only - http://bmjopen.bmj.com/site/about/guidelines.xhtml

nurses are able to recognize their problem-solving behaviour. Other research that wants to measure Lean maturity could benefit from adopting the validated scale, comprising six items from Malmbrandt and Ahlström's more detailed instrument[11], that this study found to be relevant and measurable in nursing wards.

Acknowledgement

We would like to thank Iris Brouwer, Pauline Vinks and Thomas Fransen for their contributions to the design, data collection and data analysis of the study. We are further grateful to the study participants from the hospital.

Funding

No funding has been received for this research.

Competing interests

The authors declare that they have no competing interests.

Contributions

AB contributed to the conception and design of the study, data analysis, interpretation of the data and in drafting the manuscript. AB and KA analysed the Lean leadership interview data. KA and GR contributed to the study's conception and design, and to revising the paper. PG and BM shared the scenarios from a prior study and contributed to revising the paper. KA coordinated the study. Finally, all authors read and approved the submitted manuscript.

Data sharing statement

No additional data are available.

Ethical approval

The Institutional Review Board (IRB) of the Faculty of Economics and Business has reviewed the Research Data Management Plan/Ethics checklist and came to the conclusion that 'the study entails no risk for participants, that anonymity is assured, that participants are not misled and that they are informed about their participation and have the choice to decline such participation' (reference #RDMPFEB-20180723-7173). All participants in the study gave informed consent before taking part. For this research, no approval of a medical ethical committee was required according to the criteria of the Dutch law.

References

1. Mazzocato P, Holden RJ, Brommels, M, *et al*. How does lean work in emergency care? A case study of a lean-inspired intervention at the Astrid Lindgren Children's hospital, Stockholm, Sweden. *BMC Health Serv Res*. 2012;12;28.
2. Kaltenbrunner M, Bengtsson L, Mathiassen SE, *et al*. A questionnaire measuring staff perceptions of Lean adoption in healthcare: development and psychometric testing. *BMC Health Serv Res*. 2017;17;235.
3. Gemmel P, Van Beveren S, Landry S, *et al*. Problem-solving behavior of nurses in a lean environment. *J Nurs Manage*. 2018(f.c.).
4. Burgess N, Radnor Z. Service improvement in the English National health service: complexities and tensions. *J Manage Organ.* 2012;18(5):594-607.
5. Tucker AL, Edmondson AC. Why hospitals don't learn from failures: organizational and psychological dynamics that inhibit system change. *Calif Manage Rev.* 2003;45(2):55-72.
6. Mazur LM, Chen SG. An empirical study for medication delivery improvement based on healthcare professionals' perceptions of medication delivery system. *Health Care Manag Sci.* 2009;12(1):56-66.

For peer review only - http://bmjopen.bmj.com/site/about/guidelines.xhtml

7. Aij KH, Visse M, Widdershoven GA. Lean leadership: an ethnographic study. *Leadersh Health Serv.* 2015;28(2):119-34.
8. Aij KH, Teunissen M. Lean leadership attributes: a systematic review of the literature. *J Health Organ Manag*. 2017;31(7/8):713-29.
9. Poksinska B, Swartling D, Drotz E. The daily work of Lean leaders–lessons from manufacturing and healthcare, *Total Qual Manag Bus.* 2013;24(7-8):886-98.
10. Goodridge D, Westhorp G, Rotter T, *et al*. Lean and leadership practices: development of an initial realist program theory. *BMC Health Serv Res.* 2015;15;362.
11. Malmbrandt M, Åhlström P. An instrument for assessing lean service adoption. *Int J Oper Prod Man.* 2013;33(9):1131-65.
12. Tucker AL. Workarounds and resiliency on the front lines of health care. 2009. Available from https://www.ahrq.gov/ (patient safety network AHRQ).
13. Van Rossum L, Aij KH, Simons FE, *et al*. Lean healthcare from a change management perspective: the role of leadership and workforce flexibility in an operating theatre. *J Health Organ Manag.* 2016;30(3):475-93.
14. Bass BM. Two decades of research and development in transformational leadership. *Eur J Work Organ Psy.* 1999;8(1):9-32.
15. White M, Wells JS, Butterworth T. The impact of a large-scale quality improvement programme on work engagement: Preliminary results from a national cross-sectional-survey of the 'Productive Ward'. *Int J Nurs Stud.* 2014;51(12):1634-43.
16. Karlsson C. *Researching operations management.* New York: Routledge, 2009.
17. Gioia DA, Corley KG, Hamilton AL. Seeking qualitative rigor in inductive research notes on the Gioia methodology. *Organ Res Methods.* 2013;16(1):15-31.
18. Krippendorff K. Reliability in content analysis. *Hum Commun Res*. 2004;30:411-33.
19. Mazur LM, Chen SG, Prescott B. Pragmatic evaluation of the Toyota Production System (TPS) analysis procedure for problem solving with entry-level nurses. *J Ind Eng Manag.* 2008;1(2):240-68.
20. Spear SJ. Fixing health care from the inside, today. *Harvard Bus Rev.* 2005;83(9):78-91.
21. Stone AG, Russell RF, Patterson K. Transformational versus servant leadership: A difference in leader focus. *Leadership Org Dev J.* 2004;25(4):349-61.
22. Drotz E, Poksinska B. Lean in healthcare from employees' perspectives. *J Health Organ Manag*. 2014;28(2):177-95.
23. Taher D, Landry S, Toussaint J. Breadth vs. depth: How to start deploying the daily management system for your lean transformation. *J Hosp Adm.* 2016;5(6):90-6.

Figure titles:

Figure 1 Conceptual model

Figure 2 Lean maturity levels and expected (dotted in red) and actual (blue) second-order problem-solving level per case/team

For peer review only - http://bmjopen.bmj.com/site/about/guidelines.xhtml

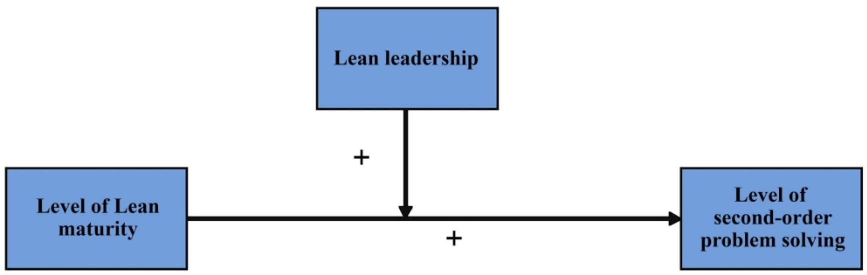

Figure 1 Conceptual model

227x126mm (300 x 300 DPI)

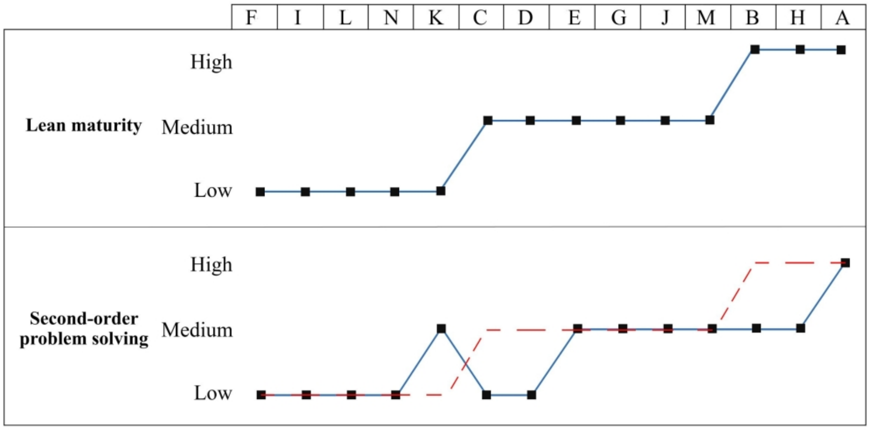

Figure 2 Lean maturity levels & expected (dotted in red) and actual (blue) second-order problem-solving level per case/team

227x126mm (300 x 300 DPI)

Reporting checklist for quality improvement study.

Based on the SQUIRE guidelines.

Instructions to authors

Complete this checklist by entering the page numbers from your manuscript where readers will find each of the items listed below.

Your article may not currently address all the items on the checklist. Please modify your text to include the missing information. If you are certain that an item does not apply, please write "n/a" and provide a short explanation.

Upload your completed checklist as an extra file when you submit to a journal.

		Reporting Item	Page Number
	#1	Indicate that the manuscript concerns an initiative to improve healthcare (broadly defined to include the quality, safety, effectiveness, patientcenteredness, timeliness, cost, efficiency, and equity of healthcare)	1, 4
	#02a	Provide adequate information to aid in searching and indexing	1
	#02b	Summarize all key information from various sections of the text using the abstract format of the intended publication or a structured summary such as: background, local problem, methods, interventions, results, conclusions	1
Problem description	#3	Nature and significance of the local problem	2
Available knowledge	#4	Summary of what is currently known about the problem, including relevant previous studies	2, 3
Rationale	#5	Informal or formal frameworks, models, concepts, and / or theories used to explain the problem, any reasons or assumptions that were used to develop the intervention(s), and reasons why the intervention(s) was expected to work	4
Specific aims	#6	Purpose of the project and of this report	2

For peer review only

		Reporting Item	Page Number
Context	#7	Contextual elements considered important at the outset of introducing the intervention(s)	4
Intervention(s)	#08a	Description of the intervention(s) in sufficient detail that others could reproduce it	4
	#08b	Specifics of the team involved in the work	4
Study of the Intervention(s)	#09a	Approach chosen for assessing the impact of the intervention(s)	4, 5
	#09b	Approach used to establish whether the observed outcomes were due to the intervention(s)	4, 5
Measures	#10a	Measures chosen for studying processes and outcomes of the intervention(s), including rationale for choosing them, their operational definitions, and their validity and reliability	4, 5
	#10b	Description of the approach to the ongoing assessment of contextual elements that contributed to the success, failure, efficiency, and cost	4, 5
	#10c	Methods employed for assessing completeness and accuracy of data	4, 5
Analysis	#11a	Qualitative and quantitative methods used to draw inferences from the data	4, 5
	#11b	Methods for understanding variation within the data, including the effects of time as a variable	5
Ethical considerations	#12	Ethical aspects of implementing and studying the intervention(s) and how they were addressed, including, but not limited to, formal ethics review and potential conflict(s) of interest	9, 10
	#13a	Initial steps of the intervention(s) and their evolution over time (e.g., time-line diagram, flow chart, or table), including modifications made to the intervention during the project	5, 6, 7
	#13b	Details of the process measures and outcome	5, 6, 7
	#13c	Contextual elements that interacted with the intervention(s)	6, 7
	#13d	Observed associations between outcomes, interventions, and relevant contextual elements	5, 6, 7

For peer review only

		Reporting Item	Page Number
	#13e	Unintended consequences such as unexpected benefits, problems, failures, or costs associated with the intervention(s).	n/a
	#13f	Details about missing data	n/a
Summary	#14a	Key findings, including relevance to the rationale and specific aims	8, 9
	#14b	Particular strengths of the project	1
Interpretation	#15a	Nature of the association between the intervention(s) and the outcomes	8, 9
	#15b	Comparison of results with findings from other publications	8, 9
	#15c	Impact of the project on people and systems	n/a
	#15d	Reasons for any differences between observed and anticipated outcomes, including the influence of context	8, 9
	#15e	Costs and strategic trade-offs, including opportunity costs	n/a
Limitations	#16a	Limits to the generalizability of the work	2, 9
	#16b	Factors that might have limited internal validity such as confounding, bias, or imprecision in the design, methods, measurement, or analysis	9
	#16c	Efforts made to minimize and adjust for limitations	9
Conclusion	#17a	Usefulness of the work	1, 8
	#17b	Sustainability	n/a
	#17c	Potential for spread to other contexts	9
	#17d	Implications for practice and for further study in the field	9
	#17e	Suggested next steps	9
Funding	#18	Sources of funding that supported this work. Role, if any, of the funding organization in the design, implementation, interpretation, and reporting	n/a

The SQUIRE 2.0 checklist is distributed under the terms of the Creative Commons Attribution License CC BY-NC 4.0. This checklist was completed on 17. September 2018 using http://www.goodreports.org/, a tool made by the EQUATOR Network in collaboration with Penelope.ai

For peer review only

BMJ Open

The role of lean leadership in the lean maturity – second-order problem-solving relationship: a mixed methods study

Journal:	*BMJ Open*
Manuscript ID	bmjopen-2018-026737.R2
Article Type:	Research
Date Submitted by the Author:	13-Mar-2019
Complete List of Authors:	Bijl, Arie; University of Groningen, Faculty of Economics and Business, Department of Operations,<br>Ahaus, Kees; University of Groningen, Faculty of Economics and Business, Department of Operations,<br>Ruël, Gwenny; University of Groningen, Faculty of Economics and Business, Department of Operations,<br>Gemmel, Paul; Ghent University, Faculty of Economics and Business Administration, Department of Innovation, Entrepreneurship and Service Management,<br>Meijboom, Bert; Tilburg University, Department of Tranzo
**Primary Subject Heading**:	Health services research
Secondary Subject Heading:	Health services research
Keywords:	Lean leadership, Lean maturity, second-order problem solving

SCHOLARONE™
Manuscripts

The role of lean leadership in the lean maturity – second-order problem-solving relationship: a mixed methods study

Arie Bijl
Department of Operations
Faculty of Economics and Business
University of Groningen, The Netherlands
Nettelbosje 2
9747 AE Groningen, The Netherlands
T. +31503637078
Email address: ariebijl94@gmail.com

Kees Ahaus **(corresponding author)**
Department of Operations
Faculty of Economics and Business
University of Groningen, The Netherlands
Nettelbosje 2
9747 AE Groningen, The Netherlands
T. +31503637078, M. +31651591859
Email address: c.t.b.ahaus@rug.nl

Gwenny Ruël
Department of Operations
Faculty of Economics and Business
University of Groningen, The Netherlands
Nettelbosje 2
9747 AE Groningen, The Netherlands
T. +31503637078
Email address: g.c.ruel@rug.nl

Paul Gemmel
Department of Innovation, Entrepreneurship and Service Management
Faculty of Economics and Business Administration
Ghent University, Belgium
Email address: Paul.Gemmel@ugent.be
Tweekerkenstraat 2
9000 Gent, Belgium
T. +3292643518

Bert Meijboom
Department of Tranzo
Tilburg University, the Netherlands
Email address: B.R.Meijboom@uvt.nl
Postbus 90153
5000 LE Tilburg, The Netherlands
T. +31134663237

For peer review only

The role of lean leadership in the lean maturity – second-order problem-solving relationship: a mixed methods study

ABSTRACT

Objectives: To investigate the relationship between Lean adoption and problem-solving behaviour in nursing teams, and to explore the practices of Lean leaders in nursing wards to reveal how they can stimulate second-order problem solving within their teams.

Design: A mixed-methods retrospective multiple case study using semi-structured interviews. Interview data were used to assess the level of Lean maturity (based on a customized validated instrument) and the level of second-order problem solving (based on scenarios). Within-case and cross-case analyses were employed to identify Lean leadership practices.

Setting: 14 nursing teams, with different levels of Lean maturity, in a Dutch hospital.

Participants: Three members of each nursing team were interviewed: the team leader, one nurse from the ward's core team for the Lean-based quality improvement programme and one nurse outside the core team.

Interventions: The nursing teams were in various phases of a Lean-based quality improvement programme: 'The Productive Ward – Releasing Time to Care'.

Results: A strongly significant positive relationship between Lean maturity and second-order problem solving was found: $\beta = 0.68$, $R^2 = 0.46$, $p < 0.001$. Further, the results indicated a potential strengthening effect of Lean leadership on this relationship. Seven Lean leadership practices emerged from the data collected in a nursing ward setting: (1) convincing and setting an example; (2) unlocking individual and team potential; (3) solving problems systematically; (4) enthusing, actively participating and visualizing; (5) developing self-managing teams; (6) sensing, as orchestrator, what is needed for change; and (7) listening, sharing information and appreciating. These practices have a strong link with transformational leadership.

Conclusions: As Lean matures, nursing teams reach a higher level of second-order problem solving. In later stages, Lean leaders increasingly relinquish responsibility by developing self-managing teams.

Keywords: Lean leadership, Lean maturity, second-order problem solving

Word Count: 4,471

Strengths and limitations of this study

- The study fills a gap in knowledge as to whether more-mature Lean teams show a higher level of second-order problem-solving behaviour.
- In addition, it provides valuable insight into typical Lean leadership practices and how these are linked to mainstream leadership theories.
- Future research attempting to measure Lean maturity in healthcare environments may benefit from using the newly developed and validated scale that incorporates six items from Malmbrandt and Ahlström's instrument.
- Another strength of the study is its use of Gioia methodology to inductively analyse the Lean leadership data.

For peer review only - http://bmjopen.bmj.com/site/about/guidelines.xhtml

- A limitation is that the nursing teams all came from one Dutch hospital, however, this choice had the benefit that it provided nursing teams with a range of Lean maturities working under similar circumstances.

Introduction

The earliest applications of Lean thinking in the healthcare sector were more than a decade ago. Since then, Lean has gained in popularity in the sector.[1] The primary goals of Lean in healthcare have been to increase the quality of care and to increase efficiency.[2] To achieve these goals, most healthcare organizations have emphasized the application of Lean tools to reduce direct waste, but neglected developing the problem-solving abilities of frontline employees.[3] This approach may have created some process improvements, but long-term hospital-wide benefits have rarely been achieved.[4]

To realize more of Lean's potential, it is often suggested that structured problem solving should be developed throughout the organization to sustainably improve processes.[3] This is inspired by Liker's 4P model that identifies four aspects of Lean: philosophy, process, people and problem-solving.[3] One well-known and effective approach to this is *second-order problem solving.* This involves the in-depth questioning of work practices to uncover and remove the root causes of problems.[5] This approach is in sharp contrast with *first-order problem solving* where problems are resolved in an ad-hoc manner, while underlying causes remain.[6] A recent study by Gemmel *et al.*[3] suggests that second-order problem solving is more prevalent than first-order problem solving in nursing teams with high levels of Lean maturity. However, evidence from a larger sample of wards is needed to confirm this.

Earlier studies have suggested that team leaders could stimulate second-order problem-solving behaviour by nurses.[5] Team leaders are crucial in creating and sustaining the benefits of Lean adoption in hospital wards since they can help to create a culture of continuous improvement, empower frontline employees and foster participation.[7, 8, 9] Although the literature often emphasizes the importance of Lean leadership, there are few empirical studies of Lean leadership in healthcare.[9, 10] Moreover, previous studies have not connected Lean leadership with other leadership theories.[9] Consequently, an in-depth empirical study of Lean leadership in nursing wards is needed, whereby the identified leadership practices can be positioned within existing leadership approaches in order to enhance our understanding of this concept.

In this paper, we study the relationship between Lean maturity and second-order problem-solving behaviour. In addition, we explore the meaning attached to Lean leadership in nursing wards and discover how Lean leadership moderates the Lean maturity – second-order problem-solving relationship. In our study of the Lean leadership concept, we focus solely on identifying leadership practices in nursing wards (i.e. in terms of team leadership). We answer the following research questions:

- How does Lean maturity affect the problem-solving behaviour of nursing teams?
- What constitutes Lean leadership in nursing wards?
- How does Lean leadership affect the relationship between Lean maturity and second-order problem solving?

Background

Lean maturity

The concept of Lean maturity needs careful consideration if one is to accurately assess the impact of Lean implementation on the problem-solving behaviour of nursing teams.[3] Malmbrandt and Åhlström[11] argue that the extent of an organization's Lean adoption can be measured using an instrument that incorporates measures to assess Lean

For peer review only - http://bmjopen.bmj.com/site/about/guidelines.xhtml

enablers, Lean practices and performance. Lean enablers represent the supporting structure or preconditions of Lean, including the training of employees and dedicating time and resources to improvement work. Lean practices correspond to Lean principles, such as continuous improvement and eliminating waste. Performance refers to the results of Lean adoption in terms of measures such as quality, customer satisfaction and costs. Together, these three dimensions determine the Lean maturity level of an organization. These can range from no adoption through the increase of continuous improvement activities to an exceptional, well-defined and innovative approach.[11]

Second-order problem-solving behaviour

Nurses, as frontline service providers, play an essential role and are in the best position to uncover and eliminate the root causes of problems and, thereby, help their team learn.[5, 6] Second-order problem solving occurs when "the worker, in addition to patching the problem so that the immediate task at hand can be completed (i.e. first-order problem solving), also takes action to address underlying causes"[6(p.63)].

Tucker and Edmondson[5(p.61)] distinguish five broad actions linked to second-order problem solving: (1) communicating to the person or department responsible for the problem; (2) bringing the problem to the manager's attention; (3) sharing ideas about the cause of the situation and how to prevent recurrence; (4) implementing changes; and (5) verifying that changes have the desired effect.

Nurses apply both first- and second-order problem-solving approaches. Quick workarounds may be needed if patients cannot wait for the promised care.[12] However, only by applying second-order problem solving can real process improvements be made. Gemmel *et al.*[3] go as far as to state that a learning organization based on second-order problem solving should be one of the ultimate goals of Lean.

Lean leadership

The importance of effective 'Lean leadership' during Lean implementations is widely recognized.[8] A few studies in healthcare have suggested that Lean leadership can be linked to transformational leadership theory.[9, 13] Under transformational leadership, followers are motivated to do more than originally expected and to feel trust, loyalty, respect and admiration towards their leader.[14] Transformational leaders develop their followers through four key dimensions. *Idealized influence* refers to a leader being a role model, one who is admired and respected by their followers. *Inspirational motivation* implies that a leader motivates those around him/her and arouses their spirit. Transformational leaders use *intellectual stimulation* to stimulate their followers to be innovative and creative. Finally, a transformational leader attends to the need for achievement and growth of individuals through *individual consideration* by acting as a coach.[14] Transformational leadership is different from transactional leadership. The latter is merely an exchange process to motivate follower compliance, where a leader clarifies performance criteria, states expectations and determines what followers receive in return.[14] The link between transformational leadership theory and Lean leadership is relatively under-researched.[9] In healthcare, there are only a few empirical studies investigating Lean leadership practices.[9, 10]

Conceptual model

Figure 1 displays the conceptual model applied in this study. First, in line with Gemmel *et al.*[3], we expect greater Lean maturity to potentially lead to an elevated degree of second-order problem solving in nursing teams. Second, since Lean leaders are seen as essential in enhancing the problem-solving abilities of healthcare staff[8], we expect

For peer review only - http://bmjopen.bmj.com/site/about/guidelines.xhtml

effective Lean leadership to strengthen the relationship between Lean maturity and second-order problem solving.

Please insert figure 1 here

Methods

This research involves 14 case studies of nursing teams in different departments of a Dutch hospital, all in different phases of a Lean-based quality improvement programme: 'The Productive Ward – Releasing Time to Care' (from now on referred to as PW). This programme trains nursing teams in how to apply Lean tools and principles in their daily work and claims to increase the time nurses have for direct patient care.[15] A key tool incorporated in PW is the Hairdryer Model. This teaches nurses how to engage in effective problem solving by discussing problems in groups, mapping the current situation, collecting data, implementing changes and assessing their impacts.

Each of the 14 nursing teams has a PW core team that leads the change. Generally, this consists of the team leader and a few nurses. To obtain reliable data, the team leader, one nurse inside and one nurse outside each PW core team were interviewed. The sample consisted of 7 males and 35 females; 14 of whom were employed for less than five years, 15 between five and ten years, 5 between ten and fifteen years, 1 between fifteen and twenty years and 7 who had worked twenty or more years in the hospital. The nursing teams came from various wards: cardiology, urology, orthopaedics, CCU, emergency department, acute admission, neurology, lung medicine, birth centre, neonatology, paediatrics, oncology, short-stay, and day nursing. Nursing teams with differing periods in the programme (0-24 months) were selected as cases. We expected this to provide us with variation on the Lean maturity variable. Cases were selected with different durations in the belief this would produce different results but for predictable reasons (enabling theoretical replication).[16] Table 1 shows the selected cases within each duration period.

Table 1 Selected cases within each duration period

Duration	Selected cases
**Period 1 (0-6 months)**	L, N
**Period 2 (6-12 months)**	H, I, J, K
**Period 3 (12-18 months)**	D, E, F, G, M
**Period 4 (18-24 months)**	B, C
**Period 5 (24 + months)**	A

Data collection

In total, 42 semi-structured interviews with nurses were conducted in December 2016 with an average duration of 40 minutes (minimum 24 minutes, maximum 68 minutes). More than one researcher was present at each interview. The interview questions related to Lean maturity were designed to measure several Lean enablers and Lean practices. These provided accurate insight into the progress being made with Lean implementation. These Lean enablers and practices were drawn from Malmbrandt and Åhlström's instrument.[11] Their instrument contains 34 items to assess Lean service adoption but we decided to include only six items for several reasons. First, some items had strong links with our other main variables, such as management commitment and understanding (Lean leadership), the degree of structured problem solving (second-order problem solving) and performance and these were excluded to avoid confounding issues between the different concepts/variables of our conceptual model. Secondly, some items were not

For peer review only - http://bmjopen.bmj.com/site/about/guidelines.xhtml

considered relevant in the hospital context, or too complex to measure (such as the levelling and balancing of workloads). These exclusions resulted in only six items being considered useful for this study. The customized instrument included three Lean enablers (employees' understanding of Lean; time and resources allocated to improvement work; and bi-directional vertical information flows), and three Lean practices (identification of patient value; workplace design for flow; and visualization of information and improvements).

Second-order problem solving was measured through a set of questions in the form of scenarios derived by Gemmel *et al.*[3] These scenarios describe several types of problems nurses face in their daily work. They were used to obtain information about the actions nurses took when faced with a problem. To obtain further insights into this concept, nurses were also asked to provide examples of a problem where they engaged in second-order problem solving.

Since the main goal of PW is to increase the proportion of time that nurses spend on direct patient care[15], the level of performance is measured by an open question asking about perceptions of PW outcomes in terms of the time that is freed up for direct patient care.

Lean leadership was studied from various viewpoints, with different sets of open and semi-structured interview questions posed to team leaders and nurses. We specifically asked about leadership practices adopted to support and implement the PW programme and to develop the team in applying PW. Questions related to generic leadership practices were avoided.

Data analysis

From each interview, scores were deduced for the levels of Lean maturity and second-order problem-solving behaviour based on the instruments applied. The scores for each set of three ward members were checked by the interviewers for consistency. This process eventually resulted, for each team, in a low, medium or high rating, for its Lean maturity and its second-order problem-solving level.

As an indicator of the construct validity of our instrument, a correlation analysis was performed to explore whether the duration of the PW programme was related to the level of Lean maturity. This revealed a strong correlation ($r = 0.58$; $p < 0.001$). Next, scores were attributed to the three enablers and three practices of Lean on a five-point scale based on the original instrument by Malmbrandt and Åhlström.[11] Because the Lean enablers and practices were extracted from a validated instrument, a factor analysis was performed to check for unidimensionality of the six selected items. Since our sample size was too small (N=42) for a confirmatory factor analysis (the model fit indices: RMSR = 0.11, RMSEA = 0.16, and TLI = 0.82 did not fully meet the criteria), a reasonable alternative consists of a two-step approach in which we first used principal component analysis (PCA) to provide evidence on the existence of only one dimension, and subsequently confirmed its internal consistency by means of a reliability analysis using Cronbach's alpha. With KMO = 0.76, Bartlett's chi-square = 92.2; $p < 0.001$, all communalities > 0.49, and explaining 56.2% of the variance, conducting a PCA was allowed. The PCA extracted a single component (Eigenvalue factor 1 = 3.37; Eigenvalue factor 2 = 0.77) with all factor loadings above 0.7, supporting the construct validity of measuring Lean maturity using the six-item scale. In addition, the reliability analysis returned a Cronbach's alpha of 0.84 suggesting a strong internal consistency among the Lean enablers and practices.

The information collected through the scenarios was used to link each respondent to a category of first- or second-order problem-solving behaviour. For this, we used a scale

For peer review only - http://bmjopen.bmj.com/site/about/guidelines.xhtml

with eight levels. Levels 1, 2 and 3 represent first-order problem-solving approaches, whereas levels 4 through 8 represent second-order problem-solving actions as described by Tucker and Edmondson[5(p.61)], with level 8 indicating the highest degree of second-order problem solving.

Since second-order problem solving is expected to enhance performance, a correlation analysis was performed to assess this relationship. The perceived performance was categorized using a four-level: (1) perception that there are no clear effects of PW; (2) perception that time is freed up for direct patient care, but only indirectly through a better organized ward; (3) perception that actual time is freed up for direct patient care and this is exemplified; and (4) time is demonstrably freed up for direct patient care.

For the within-case analysis of Lean leadership, data were extracted from all the interviews. Using the rigorous step-by-step approach proposed by Gioia *et al.*[17], the data were coded inductively by two researchers. This led to a coding tree of Lean leadership practices (available on request) involving 339 *in vivo* codes, 29 themes and 7 aggregate dimensions. The list of themes was then used by two researchers to independently code 9 out of 42 interviews (20% of the interviews). A comparison demonstrated an acceptably high inter-rater reliability in terms of assessing Lean leadership with a Krippendorff's alpha value of 0.85.[18]

In the cross-case analysis, cases where consistent low, medium or high levels of both Lean maturity and second-order problem solving were found were compared with cases that deviated from this expectation of consistency to determine whether Lean leadership strengthened the relationship between Lean maturity and second-order problem solving.

Patient and public involvement

There was no patient or public involvement in this study. The results of this work were disseminated to representatives of the hospital. Once the study has been published, the main findings will be used in the education of Master students.

Results

The results showed that increased Lean maturity positively influences the level of second-order problem solving in nursing teams. This relationship was tested through a single linear regression analysis. A strongly significant positive relationship was found between Lean maturity and second-order problem solving: $\beta = 0.68$, $R^2 = 0.46$, $p < 0.001$. There was also a strong correlation between second-order problem solving and performance ($r = 0.66$; $p < 0.001$), indicating that second-order problem solving indeed seems to free up time for patient care.

Within-case analysis

The in-depth analysis of the meaning of Lean leadership resulted in the identification of seven Lean leadership practices: (1) convincing and setting an example; (2) unlocking individual and team potential; (3) solving problems systematically; (4) enthusing, actively participating and visualizing; (5) developing self-managing teams; (6) sensing, as orchestrator, what is needed for change; and (7) listening, sharing information and appreciating. Each of these practices is explained below.

First, Lean leaders helped nurses to become acquainted with the programme. They constitute the driving force of PW by setting an example and actively promoting PW in their team. Initially, many nurses needed to be convinced of the value of PW and, so, an important activity was to explain PW modules and highlight potential benefits. These included the time that could be saved by applying PW: *"I try to give insights into why we have to change certain things"* (D-01: in this notation, D refers to team D, participant

For peer review only - http://bmjopen.bmj.com/site/about/guidelines.xhtml

'01' refers to the team leader; '02' and '03' refer to the nurses inside and outside the PW core team respectively).

Lean leaders also aimed to unlock the greater potential of nurses and their team by encouraging everyone to become active within PW, for instance, by stimulating membership of the PW core team and participation in improvement board sessions. Nurses were also invited to indicate a preferred improvement project, based on their own interests and capabilities, to work on within PW. Within these PW projects, Lean leaders adjusted their own degree of involvement for each individual based on the level of experience of each nurse. This gave room for individual potential to develop.

Further, Lean leaders were involved in systematic problem solving. They were present during team discussions where problems were analysed by applying PW tools. Leaders also encouraged their team to solve problems through the Hairdryer Model, as they felt that this model could be incorporated in nurses' work practices. In addition, the leaders cooperated with nurses who wanted to engage in systematic problem solving, as one of the participants stated: *"Our team leader would help us by providing input on how we can approach things"* (C-03).

Another key Lean leadership practice was to enthuse the team, actively participate in PW and visualize. First, the leaders encouraged nurses to organize improvement board sessions and to carry out daily evaluations of processes. We observed that core-team members were regularly rotated, as being a member of the PW core team was perceived as a strong motivating factor by nurses. This leadership involvement was seen as highly important by both nurses and leaders, as the following quotes illustrate: *"The leader motivates me through her enthusiasm, and by showing that she wants it herself"* (N-02) and: *"I need to bring along the group through enthusiasm"* (E-01). Finally, many participants pointed out that leaders used visualization through photographs or videos to create awareness of a non-optimal situation and to demonstrate PW accomplishments.

Lean leaders also developed self-managing teams. They encouraged nurses to actively take the lead, for instance, by giving nurses ownership of improving certain processes through PW tools. It was frequently noted that, as the implementation of PW progressed, Lean leaders would increasingly relinquish tasks and responsibility to increase the sense of ownership among the nurses in the team. This interpretation is supported by the following quotes: *"Through this programme, my tasks are diminished as nurses themselves take more responsibility"* (A-01) and *"The leader does not want to keep tight control of everything, he relinquishes tasks and gives guidance, which motivates us to be actively involved in the programme"* (H-03).

Lean leaders continuously thought about what was needed for change to take place. For instance, they facilitated this by providing time and resources to engage in PW activities and they ensured that PW meetings regularly took place. It was also stated that the leaders provided direction and kept an overview of PW's progress within their team, without losing track of the hospital's objectives. The leaders focused on small and manageable steps for improvement within the PW modules, carefully assessing any resistance to PW from the team and giving constant attention to ensuring the changes took place.

Finally, Lean leaders took time to listen, share information and appreciate the efforts of others. They engaged in individual conversations with nurses in order to stay involved and, if there were issues within the team, to better understand why these had emerged. Information about PW's progress was also shared actively with the team using score charts, newsletters and e-mails: *"It is my responsibility to inform them properly"* (K-01). Lean leaders also expressed appreciation to their team. Compliments were given if the team was on schedule and when certain modules had been completed successfully.

For peer review only - http://bmjopen.bmj.com/site/about/guidelines.xhtml

Cross-case analysis

Figure 2 displays the levels of Lean maturity and second-order problem solving of each team. The blue lines represent the assessed levels for each team based on the interviews, while the red line shows the expected level of second-order problem solving, based on the proposition that higher Lean maturity results in increased second-order problem solving.

The graph shows that this proposition did not hold for all the teams. In searching for patterns and reasons, cases that agreed with the proposition were compared to cases that deviated. This analysed whether the difference between actual and expected second-order problem-solving levels could be attributed to the practices of Lean leaders. More specifically, teams C and D (medium Lean maturity – low second-order problem solving i.e. 'ML') were compared with cases E, G, J, M (medium Lean maturity – medium second-order problem solving, i.e. 'MM'). Further, cases B and H (high Lean maturity – medium second-order problem solving, i.e. 'HM') were compared with team A (high Lean maturity – high second-order problem solving, i.e. 'HH').

Please insert figure 2 here

Our analysis shows that Lean leaders are able to contribute to their team achieving a high level of second-order problem solving. Second-order problem solving can be increased by leadership practices that encourage team ownership of the problem-solving process. In the HH case, team A, the leader played a crucial role in empowering and stimulating nurses to take the lead in thinking about solutions to problems and in implementing solutions through a bottom-up approach. This was explained as follows: *"She [the team leader] tells me to go and investigate how I could make a solution happen"* (A-03). In comparison, in the HM cases, the teams were self-managed to some extent, but responsibility for the entire problem-solving process had not been completely relinquished by the leaders. That is, in the latter, more involvement and active support of the leaders in the problem-solving process was observed.

Second, our analysis shows that an enthusiastic leader who actively participates in the Lean initiative as a role model can lead to a higher level of second-order problem solving. In the HH and MM teams, the leaders were seen as role models for PW through their enthusiasm and participation, as exemplified by the following quote: *"Our leader is very enthusiastic and fanatical"* (E-02). This was perceived as an important aspect that keeps PW alive in the team and, thereby, keeps nurses motivated to participate. In contrast, in the HM and ML cases, the level of enthusiasm and participation from leaders was perceived as being lower.

Discussion

The first main research question posed in this research was: *How does Lean maturity influence the problem-solving behaviour of nursing teams?* Our findings confirm the related proposition that, as nursing teams reach higher levels of Lean maturity, they also demonstrate higher degrees of second-order problem solving. As such, we add to the evidence for the existence of a positive relationship between Lean maturity and second-order problem solving.[3] This suggests that nurses may become more skilled in discovering and removing the root causes of organizational problems through Lean adoption.[19, 20] The reason is that Lean helps nurses to become acquainted with and involved in the identification, analysis and removal of the root causes of problems. In the PW programme, nursing teams were trained to use the Hairdryer Model that involves multiple activities associated with second-order problem-solving actions.[5]

For peer review only - http://bmjopen.bmj.com/site/about/guidelines.xhtml

The second research question was: *What constitutes Lean leadership in nursing wards?* In order to answer this question, Lean leadership was studied empirically in several nursing teams, resulting in the identification of seven Lean leadership practices as listed in the within-case analysis section. Overall, our findings indicate that Lean leadership in nursing wards has strong connections with transformational leadership theory, a finding in line with earlier studies.[9, 13] That is, many of the identified Lean leadership practices can be classified as transformational. For example, arousing the team spirit through motivation and inspiration are key activities in the inspirational motivation dimension.[14] Charismatic-inspirational leadership was also in evidence when Lean leaders enthused others and, at the same time, actively participated in the Lean programme themselves.[21] A further connection with individualized consideration and coaching to develop followers was observed in the Lean leaders' practice of unlocking individual and team potential. This was achieved by recognizing differences in individuals' qualities and preferences within the team.[21] Our results indicate that Lean leaders also used other transformational practices, such as listening effectively, actively sharing information and appreciating people.[21] Our results also underpin the importance of visualization in applying Lean in hospital environments.[22] Visualization was practiced by Lean leaders to help their team identify areas for improvement[9] and to demonstrate PW accomplishments on improvement boards that were located in the nursing wards.

The final research question was: *How does Lean leadership affect the relationship between Lean maturity and second-order problem solving?* The cross-case analysis revealed that successful Lean leaders increasingly relinquished responsibility for improvement activities to their team as Lean maturity increased. At the outset of Lean initiatives, large investments from leaders were required in terms of effort and resources[9] and top-down steering may initially be necessary to create the supporting structure for Lean.[23] However, in later stages, it becomes imperative to empower frontline staff so that they can take the initiative in daily improvement activities.[10] Our findings suggest that such a transition was necessary for nursing teams to reach a high level of second-order problem solving. Lean leaders have an important role in facilitating this transition by encouraging nurses to take ownership of improvement activities and by developing their team to become self-managing. The importance of stimulating a bottom-up approach to continuous improvement in healthcare is supported by existing literature.[20, 22]

Overall, our findings suggest that successful Lean implementation in nursing teams requires a bottom-up approach with responsibility for improvement activities gradually being handed over to frontline nurses, supported by leaders with strong transformational leadership skills that radiate enthusiasm towards their team. Through this, nursing teams can achieve a high second-order problem-solving level as Lean matures.

This study has a number of limitations. First, since nursing teams from only one Dutch hospital were included, the external validity of our findings could be impacted negatively. However, this choice had the benefit that it enabled us to compare nursing teams with a range of Lean maturities working under similar circumstances. Second, when we started the interview round, we realized that many respondents did not recognize all the problems provided in the scenarios of Gemmel et al.[3] This was counteracted at an early stage of data collection by asking an additional open-ended question as to whether nurses could provide an example of a problem solved through a second-order problem-solving approach. This resulted in many useful responses.

Further research could aim to improve the instrument for measuring second-order problem solving in nursing teams. This could involve adding new scenarios in which

For peer review only - http://bmjopen.bmj.com/site/about/guidelines.xhtml

nurses are able to recognize their problem-solving behaviour. Other research that wants to measure Lean maturity could benefit from adopting the validated scale, comprising six items from Malmbrandt and Ahlström's more detailed instrument[11], that this study found to be relevant and measurable in nursing wards.

Acknowledgement
We would like to thank Iris Brouwer, Pauline Vinks and Thomas Fransen for their contributions to the design, data collection and data analysis of the study. We are further grateful to the study participants from the hospital.

Funding
No funding has been received for this research.

Competing interests
The authors declare that they have no competing interests.

Contributions
AB contributed to the conception and design of the study, data analysis, interpretation of the data and in drafting the manuscript. AB and KA analysed the Lean leadership interview data. KA and GR contributed to the study's conception and design, and to revising the paper. PG and BM shared the scenarios from a prior study and contributed to revising the paper. KA coordinated the study. Finally, all authors read and approved the submitted manuscript.

Data sharing statement
No additional data are available.

Ethical approval
The Institutional Review Board (IRB) of the Faculty of Economics and Business has reviewed the Research Data Management Plan/Ethics checklist and came to the conclusion that 'the study entails no risk for participants, that anonymity is assured, that participants are not misled and that they are informed about their participation and have the choice to decline such participation' (reference #RDMPFEB-20180723-7173). All participants in the study gave informed consent before taking part. For this research, no approval of a medical ethical committee was required according to the criteria of the Dutch law.

References
1. Mazzocato P, Holden RJ, Brommels, M, *et al*. How does lean work in emergency care? A case study of a lean-inspired intervention at the Astrid Lindgren Children's hospital, Stockholm, Sweden. *BMC Health Serv Res*. 2012;12;28.
2. Kaltenbrunner M, Bengtsson L, Mathiassen SE, *et al*. A questionnaire measuring staff perceptions of Lean adoption in healthcare: development and psychometric testing. *BMC Health Serv Res*. 2017;17;235.
3. Gemmel P, Van Beveren S, Landry S, *et al*. Problem-solving behavior of nurses in a lean environment. *J Nurs Manage*. 2018(f.c.).
4. Burgess N, Radnor Z. Service improvement in the English National health service: complexities and tensions. *J Manage Organ.* 2012;18(5):594-607.
5. Tucker AL, Edmondson AC. Why hospitals don't learn from failures: organizational and psychological dynamics that inhibit system change. *Calif Manage Rev.* 2003;45(2):55-72.
6. Mazur LM, Chen SG. An empirical study for medication delivery improvement based on healthcare professionals' perceptions of medication delivery system. *Health Care Manag Sci.* 2009;12(1):56-66.

For peer review only - http://bmjopen.bmj.com/site/about/guidelines.xhtml

7. Aij KH, Visse M, Widdershoven GA. Lean leadership: an ethnographic study. *Leadersh Health Serv.* 2015;28(2):119-34.
8. Aij KH, Teunissen M. Lean leadership attributes: a systematic review of the literature. *J Health Organ Manag*. 2017;31(7/8):713-29.
9. Poksinska B, Swartling D, Drotz E. The daily work of Lean leaders–lessons from manufacturing and healthcare, *Total Qual Manag Bus.* 2013;24(7-8):886-98.
10. Goodridge D, Westhorp G, Rotter T, *et al*. Lean and leadership practices: development of an initial realist program theory. *BMC Health Serv Res.* 2015;15;362.
11. Malmbrandt M, Åhlström P. An instrument for assessing lean service adoption. *Int J Oper Prod Man.* 2013;33(9):1131-65.
12. Tucker AL. Workarounds and resiliency on the front lines of health care. 2009. Available from https://www.ahrq.gov/ (patient safety network AHRQ).
13. Van Rossum L, Aij KH, Simons FE, *et al*. Lean healthcare from a change management perspective: the role of leadership and workforce flexibility in an operating theatre. *J Health Organ Manag.* 2016;30(3):475-93.
14. Bass BM. Two decades of research and development in transformational leadership. *Eur J Work Organ Psy.* 1999;8(1):9-32.
15. White M, Wells JS, Butterworth T. The impact of a large-scale quality improvement programme on work engagement: Preliminary results from a national cross-sectional-survey of the 'Productive Ward'. *Int J Nurs Stud.* 2014;51(12):1634-43.
16. Karlsson C. *Researching operations management.* New York: Routledge, 2009.
17. Gioia DA, Corley KG, Hamilton AL. Seeking qualitative rigor in inductive research notes on the Gioia methodology. *Organ Res Methods.* 2013;16(1):15-31.
18. Krippendorff K. Reliability in content analysis. *Hum Commun Res*. 2004;30:411-33.
19. Mazur LM, Chen SG, Prescott B. Pragmatic evaluation of the Toyota Production System (TPS) analysis procedure for problem solving with entry-level nurses. *J Ind Eng Manag.* 2008;1(2):240-68.
20. Spear SJ. Fixing health care from the inside, today. *Harvard Bus Rev.* 2005;83(9):78-91.
21. Stone AG, Russell RF, Patterson K. Transformational versus servant leadership: A difference in leader focus. *Leadership Org Dev J.* 2004;25(4):349-61.
22. Drotz E, Poksinska B. Lean in healthcare from employees' perspectives. *J Health Organ Manag*. 2014;28(2):177-95.
23. Taher D, Landry S, Toussaint J. Breadth vs. depth: How to start deploying the daily management system for your lean transformation. *J Hosp Adm.* 2016;5(6):90-6.

Figure titles:

Figure 1 Conceptual model

Figure 2 Lean maturity levels and expected (dotted in red) and actual (blue) second-order problem-solving level per case/team

For peer review only

For peer review only - http://bmjopen.bmj.com/site/about/guidelines.xhtml

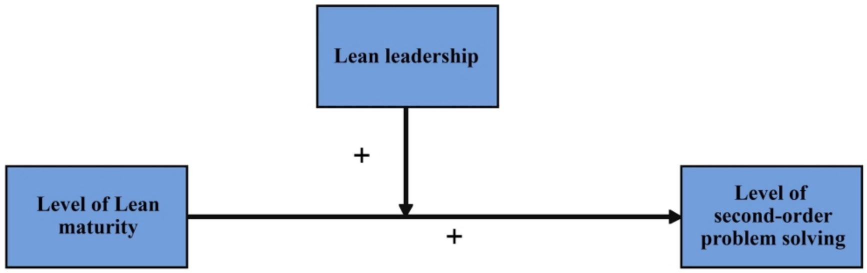

Figure 1 Conceptual model

227x126mm (300 x 300 DPI)

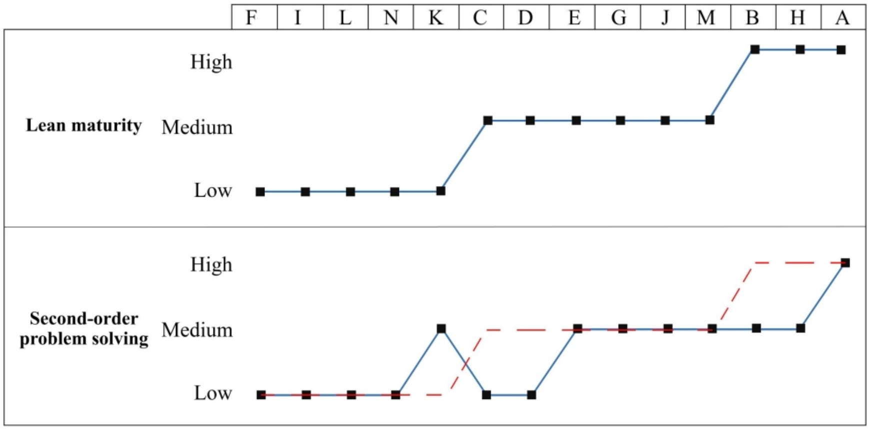

Figure 2 Lean maturity levels & expected (dotted in red) and actual (blue) second-order problem-solving level per case/team

227x126mm (300 x 300 DPI)

Reporting checklist for quality improvement study.

Based on the SQUIRE guidelines.

Instructions to authors

Complete this checklist by entering the page numbers from your manuscript where readers will find each of the items listed below.

Your article may not currently address all the items on the checklist. Please modify your text to include the missing information. If you are certain that an item does not apply, please write "n/a" and provide a short explanation.

Upload your completed checklist as an extra file when you submit to a journal.

		Reporting Item	Page Number
	#1	Indicate that the manuscript concerns an initiative to improve healthcare (broadly defined to include the quality, safety, effectiveness, patientcenteredness, timeliness, cost, efficiency, and equity of healthcare)	1, 4
	#02a	Provide adequate information to aid in searching and indexing	1
	#02b	Summarize all key information from various sections of the text using the abstract format of the intended publication or a structured summary such as: background, local problem, methods, interventions, results, conclusions	1
Problem description	#3	Nature and significance of the local problem	2
Available knowledge	#4	Summary of what is currently known about the problem, including relevant previous studies	2, 3
Rationale	#5	Informal or formal frameworks, models, concepts, and / or theories used to explain the problem, any reasons or assumptions that were used to develop the intervention(s), and reasons why the intervention(s) was expected to work	4
Specific aims	#6	Purpose of the project and of this report	2

For peer review only

		Reporting Item	Page Number
Context	#7	Contextual elements considered important at the outset of introducing the intervention(s)	4
Intervention(s)	#08a	Description of the intervention(s) in sufficient detail that others could reproduce it	4
	#08b	Specifics of the team involved in the work	4
Study of the Intervention(s)	#09a	Approach chosen for assessing the impact of the intervention(s)	4, 5
	#09b	Approach used to establish whether the observed outcomes were due to the intervention(s)	4, 5
Measures	#10a	Measures chosen for studying processes and outcomes of the intervention(s), including rationale for choosing them, their operational definitions, and their validity and reliability	4, 5
	#10b	Description of the approach to the ongoing assessment of contextual elements that contributed to the success, failure, efficiency, and cost	4, 5
	#10c	Methods employed for assessing completeness and accuracy of data	4, 5
Analysis	#11a	Qualitative and quantitative methods used to draw inferences from the data	4, 5
	#11b	Methods for understanding variation within the data, including the effects of time as a variable	5
Ethical considerations	#12	Ethical aspects of implementing and studying the intervention(s) and how they were addressed, including, but not limited to, formal ethics review and potential conflict(s) of interest	9, 10
	#13a	Initial steps of the intervention(s) and their evolution over time (e.g., time-line diagram, flow chart, or table), including modifications made to the intervention during the project	5, 6, 7
	#13b	Details of the process measures and outcome	5, 6, 7
	#13c	Contextual elements that interacted with the intervention(s)	6, 7
	#13d	Observed associations between outcomes, interventions, and relevant contextual elements	5, 6, 7

For peer review only

		Reporting Item	Page Number
	#13e	Unintended consequences such as unexpected benefits, problems, failures, or costs associated with the intervention(s).	n/a
	#13f	Details about missing data	n/a
Summary	#14a	Key findings, including relevance to the rationale and specific aims	8, 9
	#14b	Particular strengths of the project	1
Interpretation	#15a	Nature of the association between the intervention(s) and the outcomes	8, 9
	#15b	Comparison of results with findings from other publications	8, 9
	#15c	Impact of the project on people and systems	n/a
	#15d	Reasons for any differences between observed and anticipated outcomes, including the influence of context	8, 9
	#15e	Costs and strategic trade-offs, including opportunity costs	n/a
Limitations	#16a	Limits to the generalizability of the work	2, 9
	#16b	Factors that might have limited internal validity such as confounding, bias, or imprecision in the design, methods, measurement, or analysis	9
	#16c	Efforts made to minimize and adjust for limitations	9
Conclusion	#17a	Usefulness of the work	1, 8
	#17b	Sustainability	n/a
	#17c	Potential for spread to other contexts	9
	#17d	Implications for practice and for further study in the field	9
	#17e	Suggested next steps	9
Funding	#18	Sources of funding that supported this work. Role, if any, of the funding organization in the design, implementation, interpretation, and reporting	n/a

The SQUIRE 2.0 checklist is distributed under the terms of the Creative Commons Attribution License CC BY-NC 4.0. This checklist was completed on 17. September 2018 using http://www.goodreports.org/, a tool made by the EQUATOR Network in collaboration with Penelope.ai

For peer review only
